# Human–machine collaboration for improving semiconductor process development

**DOI:** 10.1038/s41586-023-05773-7

**Published:** 2023-03-08

**Authors:** Keren J. Kanarik, Wojciech T. Osowiecki, Yu (Joe) Lu, Dipongkar Talukder, Niklas Roschewsky, Sae Na Park, Mattan Kamon, David M. Fried, Richard A. Gottscho

**Affiliations:** Lam Research Corporation, Fremont, CA USA

**Keywords:** Electronic devices, Chemical engineering

## Abstract

One of the bottlenecks to building semiconductor chips is the increasing cost required to develop chemical plasma processes that form the transistors and memory storage cells^1,2^. These processes are still developed manually using highly trained engineers searching for a combination of tool parameters that produces an acceptable result on the silicon wafer^3^. The challenge for computer algorithms is the availability of limited experimental data owing to the high cost of acquisition, making it difficult to form a predictive model with accuracy to the atomic scale. Here we study Bayesian optimization algorithms to investigate how artificial intelligence (AI) might decrease the cost of developing complex semiconductor chip processes. In particular, we create a controlled virtual process game to systematically benchmark the performance of humans and computers for the design of a semiconductor fabrication process. We find that human engineers excel in the early stages of development, whereas the algorithms are far more cost-efficient near the tight tolerances of the target. Furthermore, we show that a strategy using both human designers with high expertise and algorithms in a human first–computer last strategy can reduce the cost-to-target by half compared with only human designers. Finally, we highlight cultural challenges in partnering humans with computers that need to be addressed when introducing artificial intelligence in developing semiconductor processes.

## Abstract (web summary)

A virtual process game to benchmark the performance of humans and computers for the fabrication of semiconductors leads to a strategy combining human expert design with optimization algorithms to improve semiconductor process development.

## Main

Semiconductor chips are at the core of every artificial intelligence (AI) system in the world, operating on digital 0 and 1 states defined by nanometre-sized transistor and memory cells. Fabricating these miniature devices on silicon wafers is a complicated manufacturing process involving hundreds of specialized process steps, nearly half of which require complex chemical plasma processes, such as etching and deposition^3^. Ironically, developing these critical processes that enable AI is still done by human process engineers using their intuition and experience, often turning to trial and error. The application of AI to process engineering for creating new chips is of general interest, as automation of this activity could evoke scenarios of the so-called ‘singularity’, at which AI effectively learns to build more of itself^4,5^.

AI has many examples of computer algorithms outperforming humans at complex tasks, such as playing board games such as chess and Go^6,7^. However, in these cases, the computer makes decisions only after training on or generating a large amount of inexpensive data. By contrast, collecting process data on silicon wafers is expensive: more than a thousand dollars per experiment for the wafer, plasma equipment operation and electron microscopy. Consequently, engineers typically develop semiconductor processes by testing only on the order of a hundred—out of potentially many trillions of—different combinations of plasma parameters, such as pressure, powers, reactive gas flows and wafer temperature. Unlike board games, which have clear rules, wafer-reactor systems are governed by an inestimable number of microscopic physical and chemical interactions between wafer material, plasma species and reactor parts^8,9^. The absence of sufficient data in a specific region of interest makes it difficult to form computer models with atomic-scale accuracy, known as a ‘little’ data problem^10^. Thus, the challenge we pose for AI is to reduce cost-to-target (that is, minimize the number of data needed to be collected) of developing a semiconductor process relative to an experienced human process engineer.

In this work, we benchmarked the performance of computer algorithms relative to experienced human process engineers, focusing on a scenario in which an untrained computer has access only to the data collected. Inspired by AI approaches to chess in which computer agents compete against humans, we created a process engineering game in which the goal for a player—human or a computer algorithm—is to develop a complex process at the lowest cost-to-target. Operating such a competition using real wafers would be expensive and impractical owing to uncontrolled variability from incoming wafers, metrology and processing equipment that would make it difficult to interpret the results. To overcome these practical difficulties, we operated the competition on a sophisticated virtual platform that enables benchmarking participants in the same process space.

## Virtual process game

**Fig. 1:**
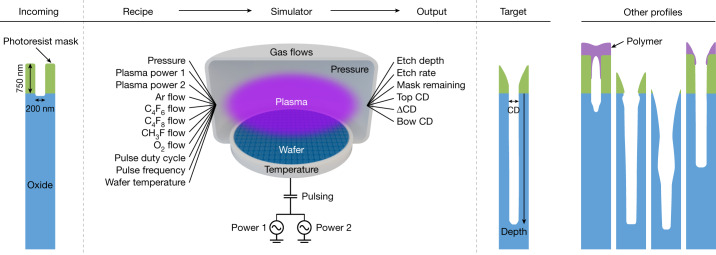
Schematic of the virtual process used in the game. The input of the virtual process is a ‘recipe’ that controls the plasma interactions with a silicon wafer. For a given recipe, the simulator outputs metrics along with a cross-sectional image of a profile on the wafer. The target profile is shown along with examples of other profiles that do not meet target. The goal of the game is to find a suitable recipe at the lowest cost-to-target. CD, critical dimension.

The competition was operated in a virtual environment designed to resemble the laboratory, as shown schematically in Fig. 1. Our case study process is a single-step plasma etch of a high-aspect-ratio hole in a silicon dioxide film, one of the many etch steps used to manufacture semiconductor chips^11^. The simulation of this process was parameterized and calibrated from existing data into a proprietary feature profile simulator, using physics-based and empirical relationships to connect an input tool parameter combination ‘recipe’ to an output etch result on the virtual wafer (Methods). To the participant, this simulator serves as an effective black-box^9^ conversion of a recipe (for example, pressure, powers and temperature) to the requirements of a process step needed to manufacture a semiconductor chip.

As in the laboratory, the goal of the game is to minimize cost-to-target of finding a recipe that produces output metrics that meet the target. The participant submits a batch (one or more recipes) and receives output metrics and cross-sectional profile images. The participant continues to submit batches until the target is met as defined in Extended Data Table 1, corresponding to the profile shown in Fig. 1. We define a ‘trajectory’ as a series of batches carried out to meet the target. Estimated from actual costs, we assign a cost of $1,000 per recipe for wafer and metrology costs and an overhead cost of $1,000 per batch for tool operation. Many potential winning recipes exist because of the high levels of degeneracy in the input parameter space. Still, we verified at the outset low odds of randomly meeting target: 0.003% per recipe based on 35,000 random samples.

## Human benchmarking

The benchmark for cost-to-target was determined by human players. The volunteers included six professional process engineers with PhD degrees in the physical sciences: three senior engineers with more than seven years of experience and three junior engineers with less than one year of experience. The engineers designed their experiments using mechanistic hypotheses based on their previous knowledge of process trends and plasma parameter dependencies. They chose an average batch size of four recipes, using univariate or bivariate parameter changes in 95% of all recipe choices. For reference, three inexperienced individuals with no relevant process experience also participated.

**Fig. 2:**
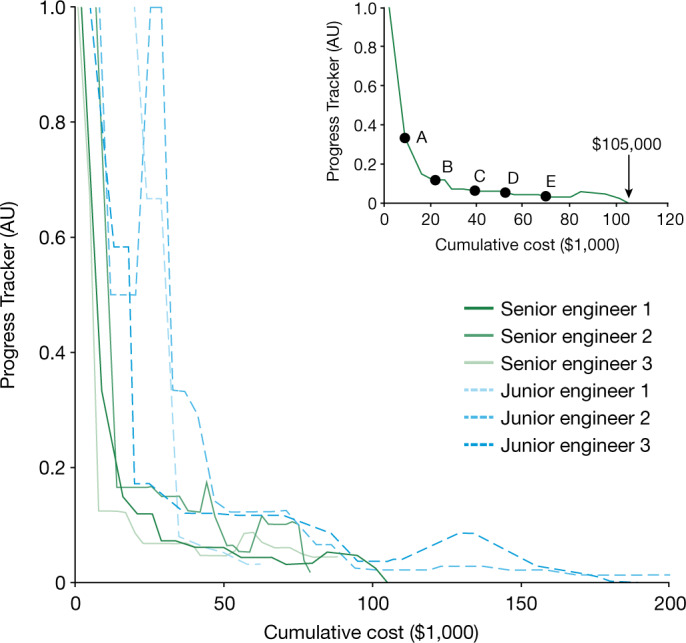
Game trajectories for human engineers. The trajectories are monitored by the Progress Tracker as defined in Methods. The target is met when the Progress Tracker is 0. Trajectories of senior engineers are in green and junior engineers in blue. The trajectory of the winning expert (senior engineer 1) is highlighted in the inset, showing transfer points A to E used in the HF–CL strategy. AU, arbitrary units. Source Data

**Extended Data Fig. 1:**
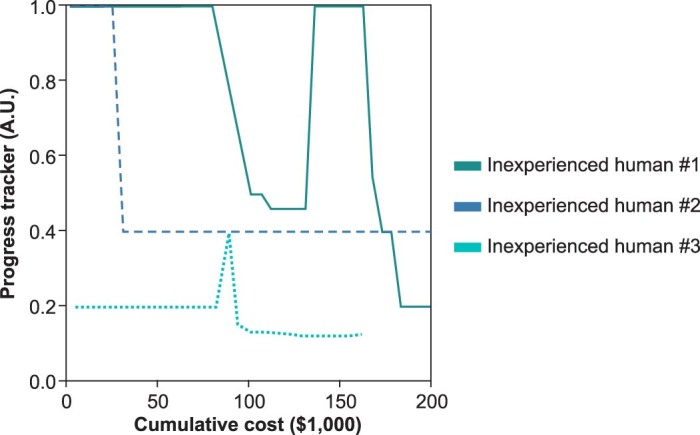
Trajectories for inexperienced humans. The target is met when the Progress Tracker is 0. None of the inexperienced participants succeeded in meeting target. Note that the cumulative cost on the *x* axis is truncated at $200,000.

Trajectories of the process engineers are shown in Fig. 2 (see Extended Data Fig. 1 for inexperienced humans and Extended Data Table 2 for a list of results). Their trajectories show qualitatively similar paths with incremental progress towards target, which we characterize into two stages: rough tuning and fine-tuning. Rough tuning refers to the initial rapid improvement towards target, whereas fine-tuning refers to the slow progress at the tail end of the trajectory at which engineers struggled to meet all output metrics simultaneously. The senior engineers required roughly half the cost of the junior engineers for the same amount of progress. The winning human participant is senior engineer no. 1 with a cost-to-target of $105,000, as shown in the inset of Fig. 2. This is our ‘expert’ human benchmark.

## Computer algorithm benchmarking

The computer algorithms participating in this competition are Bayesian optimizations—a commonly used machine-learning method for expensive black-box functions^12–14^. This class of algorithms has been studied on other applications in the semiconductor industry^15–17^. Three diverse varieties of Bayesian optimizations were selected: (1) Algo1 using Markov chain Monte Carlo sampling^18^, a multivariate linear surrogate model to compensate for the high computation cost of the sampling, and an expected improvement (EI) function. (2) Algo2 from an open-source software using the Tree-structured Parzen Estimator with an EI acquisition function^19,20^. (3) Algo3 using a Gaussian process model^21^ and a lower confidence bound acquisition function. The algorithms all use scaled Euclidean distance as the objective function and started without any training and using non-informative priors^22^.

The algorithms were programmed to use output metrics but not output profile images, and so these were effectively ignored. Only one recipe per batch was used, the default for Bayesian optimizations^23^. Trajectories were repeated 100 times for statistical relevancy to account for inherent randomness in cost-to-target owing to the probabilistic nature of Bayesian optimization. To save computational time, trajectories were truncated if they did not meet target before the expert benchmark of $105,000. We define ‘success rate’ as the percentage of trajectories with lower cost-to-target than the expert. For reference, the success rate from pure chance alone is estimated to be less than 0.2% (based on the 0.003% odds per recipe mentioned earlier).

**Fig. 3:**
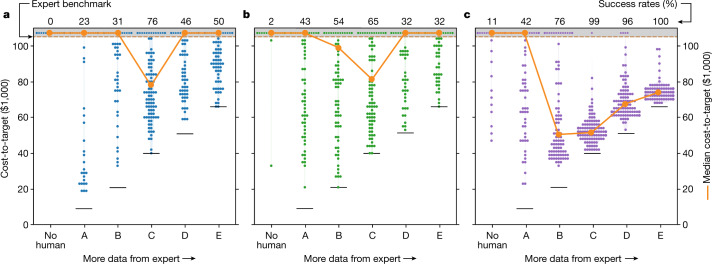
Cost-to-target using the HF–CL strategy. **a**–**c**, Results for three algorithms: Algo1 (**a**), Algo2 (**b**) and Algo3 (**c**). The ‘no human’ results are without any help from humans, as reference. Columns A to E are the transfer points shown in Fig. 2. Each dot represents one of 100 independent trajectories. Cost-to-target is the sum of cost from both the human and the computer algorithm; orange lines indicate median cost-to-target; dots aligned at the top exceed the cost-to-target of the expert alone ($105,000); black horizontal lines represent the cost of data provided by the human. Source Data

The algorithms started each trajectory with a randomly generated 32-recipe seed from a Latin hypercube, before generating the single recipe per batch. Results are labelled ‘no human’ in the panels of Fig. 3. Success rates are low, less than 1% for Algo1, 2% for Algo2 and 11% for Algo3. Altogether, only 13 out of 300 (less than 5%) attempts beat the expert. For reference, we allowed one Algo2 trajectory beyond the truncation limit, eventually meeting target at $739,000, nearly an order of magnitude more costly than the expert. Overall, the algorithms alone failed—badly—to win the competition against the human expert.

## Human first–computer last strategy

We suggested that the algorithms failed because they wasted experiments navigating the vast process space with no previous knowledge. By contrast, we speculated that process engineers drew on their experience and intuition to make better decisions in their initial navigation. Therefore, we decided to test a hybrid strategy, in which the expert guides the algorithms in a human first–computer last (HF–CL) scenario. In this implementation, instead of random sampling, the expert provides experimental data collected up to a transfer point labelled A to E in Fig. 2 (also defined in Extended Data Table 3), along with search range constrained by the expert (Extended Data Table 4). For reference, the success rate for finding the target in this ‘constrained’ search range is estimated to be 13% based on a 0.27% per recipe chance of meeting target on 2,700 random samples. In the HF–CL strategy, once the computer takes over decision-making, the expert effectively relinquishes control and has no further role in experimental design. As before, for statistical relevancy, each condition was repeated 100 times.

In the HF–CL strategy, transfer point A provides the least amount of data from the expert to the computer algorithm. At this point, the median cost-to-target for HF–CL is still consistently higher than the expert alone, with a success rate of only 20% for Algo1, 43% for Algo2 and 42% for Algo3. Although these values are substantially higher than the computer-only results, success rates of less than 50% indicate that costs are more likely to increase than decrease. Thus, although some initial guidance has improved the computer algorithm performance, HF–CL statistically fails at point A.

Figure 3 shows HF–CL results with progressively more data provided to the computer algorithm. We observe a V-shaped dependence of cost-to-target on the amount of expert data. From points A to C, access to more expert data reduces the overall cost-to-target as the algorithm performance improves. However, the trend reverses beyond point C, at which access to more expert data adds cost without clear benefit to the algorithm. The optimal performance of HF–CL for all algorithms is at point C. Algo3 greatly outperforms the other algorithms, attributed to either the flexibility of Gaussian process models or its different acquisition function, as the lower confidence bound algorithm has been shown to outperform the EI function^23^. HF–CL with Algo3 sets a new benchmark, with a median cost-to-target of $52,000—just under half the cost required by the expert alone.

**Extended Data Fig. 2:**
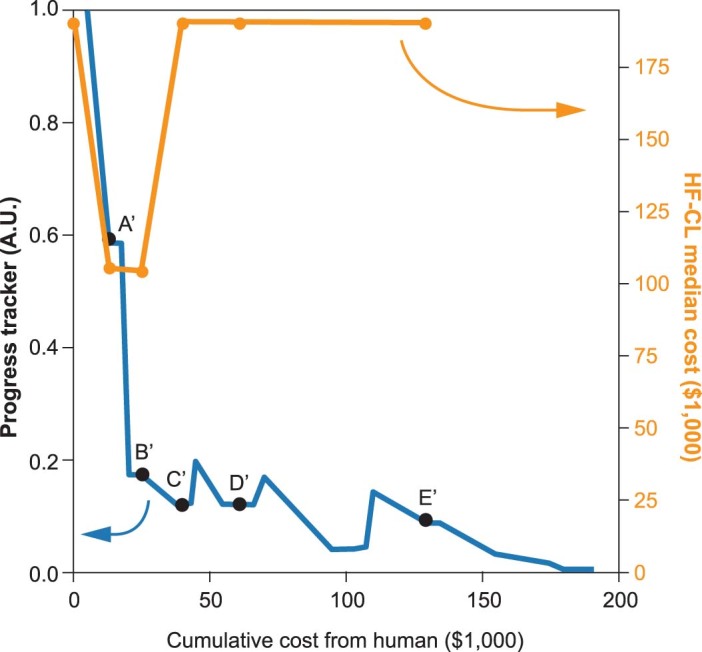
HF–CL results with junior engineer. The blue line is the trajectory of junior engineer #3 alone, with cost-to-target of $190,000. The orange line on the secondary axis is the median cost of HF–CL (using Algo3) at the transfer points indicated (Extended Data Table 3). The V-shaped dependence of cost-to-target is apparent. HF–CL provides an impressive cost savings at point B′ compared with the junior engineer alone. However, the cost-to-target is still markedly higher (about double) than HF–CL using the expert–Algo 3 combination.

**Extended Data Fig. 3:**
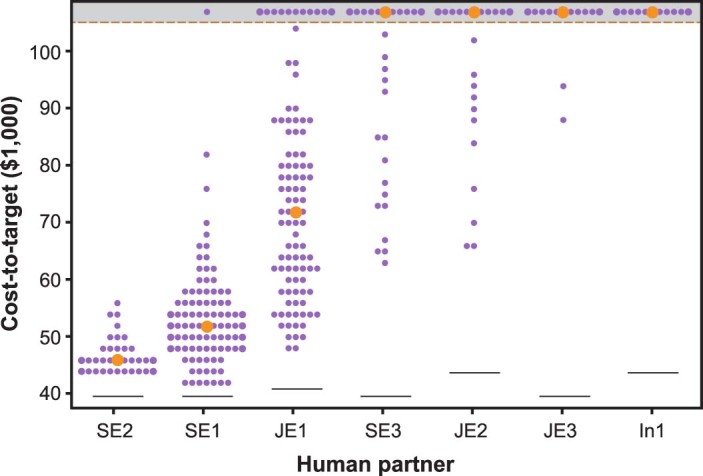
HF–CL results with different humans. Results for different humans partnered with Algo3 in the HF–CL strategy. See Extended Data Table 2 for notation. (Note that SE1 is point C in Fig. 3c and JE3 is point C′ in Extended Data Fig. 2.) Each human transferred an equivalent of $40,000 of data (or nearest full batch; see Extended Data Table 5) along with a constrained search range (Extended Data Table 4) to the computer. As In1 did not have enough experience to constrain the range, an adaptive range parameter searching 10% beyond the data distribution was used. Each dot represents one of 100 independent trajectories. Cost-to-target is the sum of the cost from both the human and the computer; orange lines are median cost-to-target; black horizontal lines indicate cost transferred from the human. The lowest costs are obtained with the highest experience levels. Overall, the results support that the HF–CL strategy is more effective at lowering costs when partnered with more experienced humans.

**Extended Data Fig. 4:**
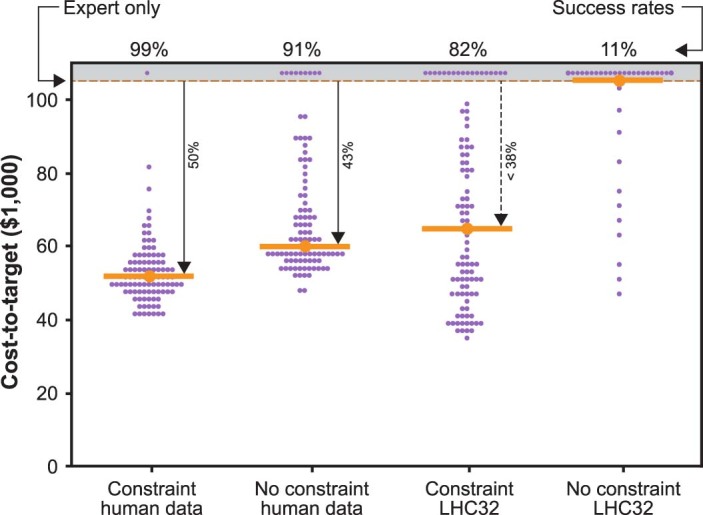
HF–CL results without constraints or without human data. This figure shows results for HF–CL using the expert and Algo3. The first column is point C in Fig. 3c, in which Algo3 is provided with both 32 points of expert data and the constrained range. In the second column, Algo3 is provided with only the expert data but not the constrained range (instead using an adaptive range parameter searching 10% beyond the data distribution). In the third column, Algo3 is provided with the constrained range but no data from the expert, instead using 100 different 32-point Latin hypercube (LHC) random sampling seeds. In the fourth column, Algo3 is provided with no information from the human. The black arrows show % cost savings relative to the expert alone, with a dashed arrow in the third column because we did not charge for access to the constraint. Each dot represents one of 100 independent trajectories. The performance of Algo3 with both the expert constrained range and the human data suggests that the engineer, if possible, should provide both data and the constrained range when implementing HF–CL.

Thus, the HF–CL strategy using the expert partnered with Algo3 won the game, by reliably reducing the cost-to-target of developing the plasma etch process relative to the expert benchmark. (See Extended Data Figs. 2 and 3 for HF–CL results with other humans and Extended Data Fig. 4 for HF–CL results without the constrained range.)

## Interpretation

The virtual process environment provides a means of testing different approaches to process development in the semiconductor industry, an activity that would have been prohibitively costly in the real laboratory. The performance of humans across different skill levels—from experts to novices—provides qualitative points of comparison on the same process. The results show that senior process engineers develop processes at about half the cost-to-target of junior process engineers, indicating the importance of domain knowledge in our industry. The computer algorithms, lacking any previous training, showed poor performance relative to the expert, with fewer than 5% of all their trajectories meeting target at lower cost-to-target. This confirms our initial expectation that computers starting from scratch will fail—they can meet the target, but at too high a cost. This is the little data problem manifested. We simply cannot afford the amount of data required for a computer to accurately predict a process recipe.

A key result of this study is the success of the HF–CL strategy. This strategy relies on an expert having the advantage early in process development and the computer algorithm excelling in the later stage. By combining these advantages, HF–CL was shown to reduce cost-to-target by half relative to the expert alone. The advantage of the human expert is attributed to the importance of domain knowledge, which these algorithms lacked, to qualitatively navigate the seemingly boundless possibilities of recipe choices. It might be intuitive that human guidance helps computers, but if algorithms are better at dealing with massively large complex problems, presumably they could have dominated at the beginning of development^24^. Instead, the computer algorithms became competent only after relevant data were provided and, preferably, with a constrained range as well. The principle of HF–CL is reminiscent of early efforts on other AI problems, suggesting that it could be generalizable to other little data problems. For example, in the beginning of computer chess (before big data), the first program in 1951 was deployed for only the last two moves, whereas opening moves remain largely the same as those determined by humans^6^. In protein folding, the Nobel Prize technique of directed evolution also requires a ‘suitable starting point’ provided by humans^25^.

Although HF–CL might seem obvious in retrospect, the results show that it only works under certain circumstances. Even with the benefit of partnering with an experienced engineer, the success of HF–CL also depends strongly on when the human transfers to the computer: if too early, the algorithms do not have sufficient guidance; if too late, the human becomes a cost burden. This principle is embodied in the convex V-shaped cost-to-target dependence on more expert data in Fig. 3. Our interpretation of the V shape is that the depth represents the maximum cost savings relative to the expert, whereas the vertex represents the optimal transfer point from human to computer. The left side of the V corresponds to improved performance of the algorithms with more data. This portion of the V is consistent with previously reported observations and the general notion that more data is better^10^.

The more unusual and notable part of the V is the right side. This is where cost-to-target rises even as the algorithms obtain access to more expert data. Here the high cost of data has led to a cost penalty for poor recipe choices by the human, illustrating the importance of the quality of data. The value of intuition even for our experienced senior engineer has markedly diminished, enabling the computer algorithms to become statistically more competent at choosing recipes. The overlap of the inverted regime with the fine-tuning stage suggests that this stage may be better relegated to computer algorithms. The observation of the V-shaped phenomenon for different human and computer combinations strengthens our belief that our insights are generalizable to this little data problem, despite the relatively small number of test cases. Furthermore, we believe that the V-curve phenomenon is a natural consequence of trying to minimize cost in the limit of expensive data and tight tolerances—as is the case in many manufacturing processes—when the need for more data directly competes with the cost of obtaining that data.

For the industry to implement the lessons of the HF–CL strategy to real semiconductor processes, it will be essential to understand how the insights apply to other processes and when humans should give up control—namely, how to identify the ideal transfer point ahead of time. We showed that the cost savings depends on the specific human–algorithm combination (Fig. 3 and Extended Data Figs. 2 and 3). Furthermore, we expect that the right side of the V might not be apparent if targets were relaxed or, conversely, might dominate in processes that only need retuning, such as in chamber matching (or transferring a process to another tool). Human knowledge may be particularly important in a high-dimensionality exploration space, effectively delaying transfer to the computer. Other factors that might affect the transfer point include process noise, process drift, target tolerance, batch size, constrained range and cost structure. We have much to learn. These topics are good candidates for further systematic study on the virtual process platform.

**Extended Data Fig. 5:**
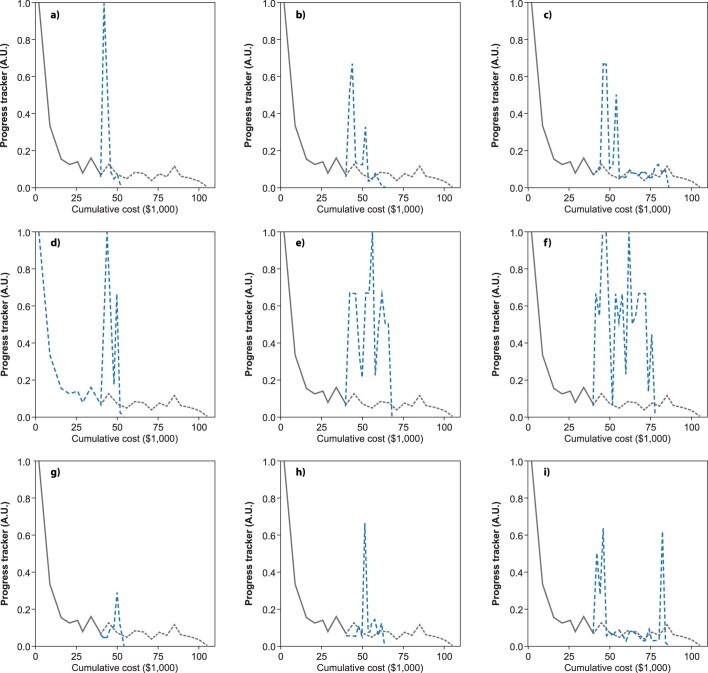
Samples of HF–CL trajectories. The target is met when the Progress Tracker is 0. The expert (SE1) trajectory is shown in grey, with transfer to the computer at point C. The blue line is the trajectory of the algorithm; the dashed grey line is the continuation of the trajectory for the expert only. The algorithm is Algo1 in panels **a**–**c**, Algo2 in panels **d**–**f** and Algo3 in panels **g**–**i**.

Beyond technical challenges, there will also probably be cultural challenges in partnering humans with computers^26,27^. In our study, we observed computer behaviour at odds with how process engineers usually develop processes. (1) The engineers almost exclusively used univariate and bivariate parameter changes to rationalize their experimental design, whereas the computers used multivariate parameter changes without any explanation. Humans may find it difficult to accept recipes that they do not understand. (2) The engineers requested an average of four experiments per batch, whereas the computers were limited to only one experiment per batch—which is probably viewed as inefficient in the laboratory. (3) Engineers steadily progressed towards target (Fig. 2), whereas the computers used exploratory recipe-choice strategies that seem sacrificial (Extended Data Fig. 5). Counterintuitive and unemotional moves are well documented in game-playing by computers^28^. In the laboratory, process engineers will need to resist intervening and inadvertently raising costs—without any guarantee of success. Ultimately, trusting computer algorithms will mean changing decades of cultural expectations in process engineering. We hope that the virtual environment will help process engineers to better understand how to partner with computers in developing process technologies.

## Outlook and conclusion

The application of AI to process engineering is still in its infancy. Human expertise will remain essential for the foreseeable future, as domain knowledge remains indispensable in navigating the earlier stages of process development. Yet, the success of the HF–CL strategy is showing us that humans, as in previous automation applications, will soon be relieved of the tedious aspects of process development. In the future, computer algorithm capability could be enhanced by encoding domain knowledge into the algorithms (either explicitly or indirectly) to enable earlier transfer points. There is rich literature on domain transfer learning, in which data from similar but not identical domains may be harnessed to accelerate learning in new domains^29^. Another area of interest in the AI field is imprinting domain knowledge in the form of a previous belief^23,30^. Indeed, creating or learning a good prior might be considered competition to the HF–CL strategy studied here. Other potential approaches in the literature include incorporation of mechanistic physics models^10^. In any case, the highly nonlinear and complex relationships between input and output parameters mean that more data will be needed to update any previous model in the vicinity of the target, in which higher-order interactions become prominent. The perpetual need for more data in specific regimes of interest practically guarantees that process engineering will continue to be susceptible to the little data problem even with the help of computer algorithms.

In summary, although computer algorithms alone could develop a process independently by using large amounts of data, they failed to do so at lower cost-to-target than the human benchmark. Only when partnered with an expert to guide towards a promising regime could the algorithms succeed. The results of this study point to a path for substantially reducing cost-to-target by combining the human and computer advantages. This unconventional approach to process engineering will require changes in human behaviour to realize its benefits. The results of this study strengthen our confidence that we are on the path to changing the way processes are developed for semiconductor chips in a marked way. In doing so, we will accelerate a critical link in the semiconductor ecosystem, using the very computing power that these semiconductor processes enable. In effect, AI will be helping to create itself—akin to the famous M. C. Escher circular graphic of two hands drawing each other.

## Methods

### Creation of the virtual process

The testing platform represents a typical engagement in our industry in which input parameters are chosen to meet target specifications provided by the semiconductor manufacturer for stringent performance criteria. Simulated tool parameters and ranges (‘Unconstrained’ values in Extended Data Table 4) are based on a generic dual-frequency plasma etch reactor^31^. Output metrics are obtained from the simulated profile.

For each chosen recipe, participants are given six output metrics along with a simulated SiO_2_ hole profile. For the output metrics, CD denotes ‘critical dimension’. Top CD is measured at the top of the SiO_2_ hole, whereas ΔCD (top CD − bottom CD) is calculated by subtracting the width at 90% of the etch depth (‘bottom’) from top CD. Bow CD is synonymous with the maximum width of the feature. Mask height refers to the height of the photoresist mask designed to protect the underlying material from etching. The initial photoresist mask height is 750 nm and the initial CD is 200 nm in diameter.

It is worth noting that process time is not an input parameter because we simulate an etch depth detector to automatically stop the etch at the desired depth. To save computational time, the simulation is stopped if too much polymer deposits on top, CDs become too wide or the etch rate is too slow. The etch rate is calculated from post-etch depth divided by (virtual) time-to-end point.

Input parameters control plasma creation in the chamber above the semiconductor wafer. Plasma ignition turns incoming neutral gases into a complex mixture of ions, electrons and reactive radicals that impinge on the wafer. Process chemistry and input parameters used are typical for plasma etching of SiO_2_ (ref. ^32^). Radiofrequency powers ignite the plasma and modulate the ion energy and angular distribution functions. Fluorocarbon gases (C_4_F_8_, C_4_F_6_ and CH_3_F) control the SiO_2_ etch by balancing the formation of volatile compounds, such as SiF_4_, CO and CO_2_, and deposition of a Teflon-like passivation layer to protect the mask and sidewalls^33^. Fluorocarbons and O_2_ flow parameters provide other means to increase or decrease carbon passivation, respectively. The etched profile is produced from the time evolution of ion and radical fluxes interacting with the materials on the wafer surface and by calculating how the etch front evolves with time.

We use a proprietary feature profile simulator, a substantially augmented version of the commercial SEMulator3D process simulator from Coventor^34^. The version we use models the detailed physical and chemical processes occurring during etching, using plasma and materials parameters such as ion yield, ion flux and reactive sticking coefficients. We transform the 11 input parameters into a dozen plasma and material parameters for the profile simulator. Whenever possible, we use established principles, derived from kinetic theory of gases and the Arrhenius equation, to transform input parameters such as pressures and wafer temperatures to fluxes and reaction rates. When available, we use empirical relationships from the literature^35–37^ plus proprietary diagnostic measurements.

SEMulator3D uses a variety of computational methods, including discrete voxel operations, and both static and transient level-set methods^38^. The central model in this publication uses a transient level-set method with a proprietary flux-based high-fidelity plasma physics model. In the level-set method, there is no explicit representation of the points on the surface. Instead, the distance from the surface is stored as a distance field based on the volume around the structure rather than the surface. A partial differential equation is then solved in the volume to propagate the distance field through time, using speed *r* *=* *r*(*x*, *t*) (which represents etch, sputter and deposition rate) of the surface motion, suitably extended to be a volume quantity. The primary cost of computing *r*(*x*, *t*) at any instant of time is the computation of particle fluxes to each point on the profile surface. These fluxes differ from those provided by the plasma model owing to both shadowing inside a deep feature and particle reflection after collision with other points on the surface. In particular, the flux at a point *x* is computed as an integral over the surface of the portion of the particle density *f*(*x*, *v*) impinging on the surface, in which *v* is the velocity^39^. The flux-based level-set methodology is in contrast to a pseudoparticle method, which tracks a pseudoparticle through its lifetime from the plasma until it reacts and changes the chemical contents of a mesh cell in the model^40^.

To compute the simulated profiles in this publication, the flux integral was estimated numerically to compute the speeds *r* *=* *r*(*x*, *t*), which were then used in the finite difference scheme to solve the level-set partial differential equation^38^. To save computation time, we chose a large spatial discretization of 25 nm, which leads to an observed variability of ±2 nm on a typical run. Each simulation takes less than ten minutes using 16 central processing units cores and 32 GB of RAM.

The process test platform was iteratively cross-validated and adjusted until it qualitatively reproduces experimental recipe data from high-aspect-ratio contact applications. Sensitivity analysis was used to investigate deviations with every input parameter to ensure the model agrees with known trends.

The inner program of the process test platform was not divulged to humans tasked with solving the process challenge nor to the data scientists developing AI optimization algorithms. This was done to prevent any possible result biases or reverse engineering of our platform.

### Calculation for Progress Tracker

The Progress Tracker is our performance indicator for monitoring how close a process is to target. To clarify, this indicator is only to illustrate progress; it was not shown to any participants or used by any computer algorithms. In practice, process engineers monitor progress to target using a ‘control table’ in which process outputs, such as etch rate, are colour-coded depending on whether they met target, were close to target or failed to reach target. There is no standard single-value performance indicator to represent this entire table, so we designed the Progress Tracker for this purpose. Our Progress Tracker has values from 0 to 1 depending on whether the process met spec (0), fails (1) or is somewhere in between (0–1). We classify etch stop and mask consumption as failures (1).

To calculate the Progress Tracker, we take the mean of six scores from the six output metrics, normalized to 1, using the definitions in Extended Data Table 1. Each output metric is assigned a score of 0 if it meets the target values. (All values must have a score of 0 for the process to meet target.) An output metric is assigned a score of 1 if it is far from target. For output metrics that are close to target, the score was decreased linearly from 1 to 0. The Progress Tracker gives a score of 1 if the process fails because of etch stop (etch depth less than 2,000 nm) or if no mask remains (‘mask remaining’ equals 0). Once Progress Tracker values are computed for every experiment, the Progress Tracker is then plotted as the best score per batch with a rolling window of four batches in Fig. 2 and Extended Data Fig. 1 and one batch in Extended Data Fig. 5.

## Online content

Any methods, additional references, Nature Portfolio reporting summaries, source data, extended data, supplementary information, acknowledgements, peer review information; details of author contributions and competing interests; and statements of data and code availability are available at 10.1038/s41586-023-05773-7.

## Supplementary information

Peer Review File

## Source data

Source Data Fig. 2

Source Data Fig. 3

## Figures and Tables

**Extended Data Table 1:**
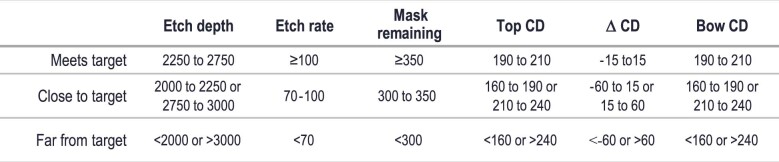
Process output targets

This table shows the output metrics that meet target and the values used in the calculation of the Progress Tracker. All units in nm except etch rate in units of nm min^−1^. CD, critical dimension; ΔCD, difference between the top and bottom CD of the feature.

**Extended Data Table 2:**
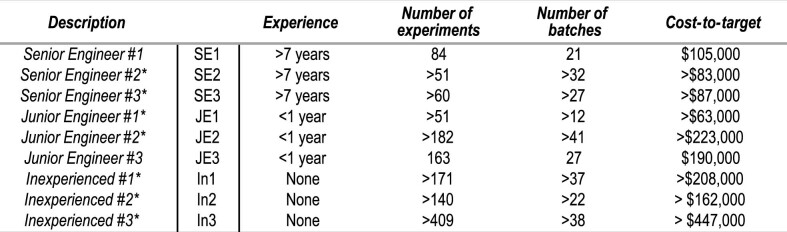
Results of all human participants

The ‘expert’ is senior engineer #1. All engineers had PhD degrees. In, inexperienced participant; JE, junior engineer; SE, senior engineer. The inexperienced humans were not process engineers.

*Participants did not meet target.

**Extended Data Table 3:**
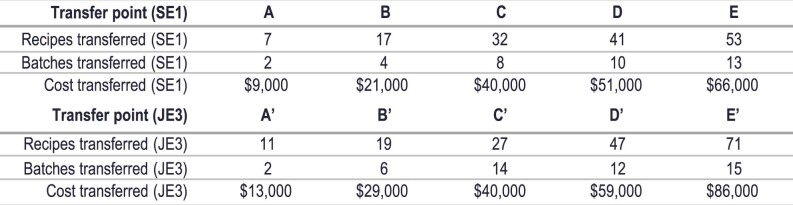
Transfer points used in the HF-CL strategy

This table shows the recipes, batches and costs for transfer points used in the HF–CL strategy with the expert (SE1) and junior engineer #3 (JE3).

**Extended Data Table 4:**
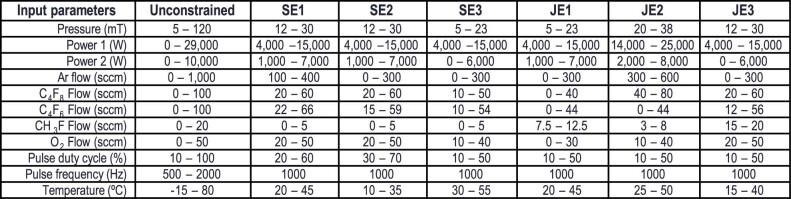
Input parameter search ranges

Unconstrained range along with the constrained ranges used in the HF–CL strategy. The constraints reduce each parameter by roughly one-quarter to one-half of the original range. All constrained ranges are the same size to simplify comparison; ranges differ as humans explored different regimes. sccm, standard cubic centimetres per minute.

**Extended Data Table 5:**
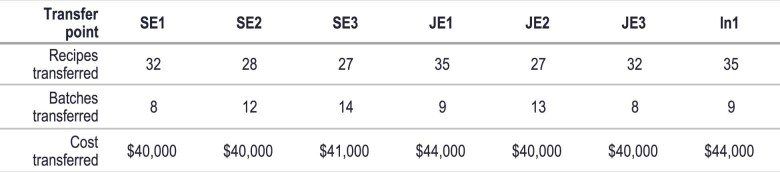
Transfer points for Extended Data Fig. 3

This table shows the recipes, batches and costs transferred in the HF–CL strategy in Extended Data Fig. 3. The abbreviations for the human participants are defined in Extended Data Table 2.

## Data Availability

Source data for Figs. 2 and 3 are provided with the paper. Further data that support the findings of this study are available from the corresponding author on reasonable request.

## Code availability

Demonstration of the simulation software used in this study, which operates on an internal platform, is available from the corresponding author on reasonable request.
