# Supplementary material for: Human–machine collaboration for improving semiconductor process development
Source: Nature. 2023 Mar 8;616(7958):707–11. doi: 10.1038/s41586-023-05773-7 (PMC10132970; doi:10.1038/s41586-023-05773-7)
Supplement: Supplementary file 1 — Peer Review File [file 41586_2023_5773_MOESM1_ESM.pdf]

Peer Review File

Manuscript Title: Humans partnering with AI to create semiconductor processes

Reviewer Comments & Author Rebuttals

Reviewer Reports on the Initial Version:

Referee #1 (Remarks to the Author):

A. Summary of key results

The paper argues that combined human expert and AI algorithms have benefits for design of complex semiconductor process recipes, compared to a “from scratch” algorithm alone, or human expert alone. The methodology of the paper is to perform and analyze results from a challenge problem – to design a plasma etch process (find process recipe settings for 11 process parameters) that achieves within-specification results across six process performance metrics. Virtual fabrication process runs, with associated per-run and per-batch fabrication costs, are available to the designer (human and/or algorithm) in order to explore the design space and corresponding results. The goal for each designer is to meet specifications with as low a total fabrication cost as possible. The experimental results presented demonstrate substantially better (about 50% lower) costs for the combined “human first – computer last” (HF-CL) approach, than for the best performing experienced senior engineer expert alone.

B. Originality and significance

The results are interesting, original, and relevant to the semiconductor technology community and beyond. The paper will be a valuable contribution to the field if key additional information, data, and issues can be addressed in revision of the paper.

C. Data and methodology

Several important areas or issues related to the methodology need additional information, in order for the reader to understand and be convinced by the baselines and comparisons presented. First, more clarity is needed about what information and methods the human designers had access to, for their design explorations. In Fig. 1, cross sections of etch features are shown; did the human experts have access to these plots, or only the values for the six output metrics? In other words, is exactly the same information provided to both the humans and the algorithms? More importantly, are there restrictions on methods the humans could use (or, conversely, more description of the methods that the humans actually did use, should be included)? The paper refers to “experience and intuition,” and also to (presumably conventional engineering) training to use single or two-parameter at-a-time exploration. But well-trained engineers will typically employ conventional design of experiment (DoE) strategies, usually in conjunction with basic response surface modeling, regression, and statistical tools. Were the engineers allowed to use these basic engineering methods? Did they? Or were they restricted to just selecting conditions and observing outputs, without pencil, paper, spreadsheet, statistical package, or other tools? Or (hopefully and more persuasively) if they did

have access to such methods, a description of what conventional tools they used can be included in the paper, so that the reader would understand that engineers struggle, even when using readily available and conventional engineering methods, in this kind of complex design problem.

Second, and in a related fashion, some additional discussion about the Bayesian optimization baselines is needed. The paper is clear that, for algorithm-only design trajectories, each start with 32 Latin hypercube experiments (and provides the range for parameters based on equipment limits). Bayesian optimization (BO) methods, however, also explicitly have some formulation of a prior belief, and it would help to mention or disclose those. Perhaps these are "non-informative" priors? Such limited prior belief approach is appropriate for the paper; however, the authors should note that this might be the most "difficult" starting scenarios for such algorithms. In practice, there is great interest in the field in imprinting some domain knowledge in the form of a good starting or prior belief (e.g. V. Fortuin, "Priors in Bayesian Deep Learning: A Review," arXiv, May 2021). Indeed, creating or learning a good prior might be considered competition to the HF-CL approach, or certainly is an area/approach worth mentioning as future research.

The methodology for HF-CL hybrid optimization needs a some clarification. For the different "transfer" points from A to E, what information and exactly when is that information transferred to the BO algorithm? It's clear from the tables which experimental run data is transferred, but it's not clear when the narrower "Expert constraints" (Table S2) are transferred to the BO algorithm. Is that starting at point A? Do these change or get tighter from A to B to E?

This clarification about runs vs. constraints is especially important, because some statements are made in the paper that need better experimental explanation, or additional experiments, to better understand. In some places, the paper suggests it is too large an exploration space that challenges the BO algorithms (e.g., "the computational algorithms only became competent after the search space was simplified"), and elsewhere it is the lack of data ("little data") that limits the algorithms. Because BOTH additional experimental runs AND restricted parameter ranges are provided in A through E cases, it is hard to decouple these effects. What would have happened in the following cases: (1) only the restricted expert-provided parameter ranges were provided to BO; (2) only the specific runs but no change to the exploration space are provided to BO; vs. (3) providing both, as was done in the paper. Adding cases 1 and 2 to the experimental dataset would help decouple these issues and provide better justification for statements in the paper.

The paper has an extended discussion of the V-shaped cost vs. amount of run information curves, for the combined HF-CL approach. It is stated in the paper that "the vertex for all algorithms in Fig. 3 corresponds to the inflection to the fine-tuning stage." This might be true for the case shown in Fig. 3, for the one succeeding senior engineer. But this does not seem to be the case for the one succeeding junior engineer, as shown in Fig. S3, where point A (quite far from "fine tuning") is very nearly the same as point B, in cumulative cost. So it may be that the paper discussion of the V shape is too narrowly dependent on the "best expert" experiments that were run. Indeed, a good (junior) domain expert combined with the BO GP algorithm seemed to achieve impressive benefits, just by getting the algorithm started even with perhaps $20-30K of experiments. Related to Fig. S3, it is not clear if the junior engineer also provided narrower parameter exploration ranges to the BO algorithm? If so, those should be added as a column in Table S2.

In terms of data and methodology, having Fig. S3 is a great help – i.e., seeing the HF-CL median cost for the (succeeding) junior engineer helps contrast with the curve for the (succeeding) senior engineer. It would also be informative to show similar plots for the other two senior engineers and other two junior engineers; even though they did not succeed on their own in meeting specifications, it would bolster or shed light on the argument about HF-CL being a good approach, to elucidate if the approach works also in conjunction with other human designers (and not just in combination with engineers who were able to meet the specifications on their own). Indeed, it might even be interesting to see a similar "V" curve (or lack thereof) for the lay-person cases – to better make the case that domain expertise helps or is needed as part of the HF-CL approach and buttress the claim that "domain knowledge remains indispensable in navigating the earlier stages of process development."

A small side comment is also worth mentioning, or suggesting, for Table S2. It is hard for the reader to get a sense of how big the solution (not the exploration) space is for this design problem. In other words, maybe there are many combinations and ranges of process parameters that achieve acceptable results? Or is there a relatively small space, in the end, where the process recipe has to sit, in order to meet specifications? One way to convey this might be to add another column to Table S2, that shows, across all successful recipes, what the ranges in each of the 11 process recipe parameters were (e.g., perhaps we find that O2 flow ALWAYS had to be in the 22 to 23 sccm range for a recipe to work.)

Fig. S5 shows three different examples of HF-CL trajectories, in support of the claim that the BO algorithmic trajectories are often quite different than the progressively improving human expert trajectories. However, this plot is shown for the TPE algorithm, which was the worst performing of the three BO approaches in Fig. 3. It would be better to show sample trajectories for the BO GP algorithm, since that was presented as the best or winning approach (and avoids the reader wondering if the trajectories of Fig. S5 are due to, and a manifestation of, it being an inferior algorithm in this case).

For the BO optimization, after the first 32 runs (as well as after the transfer from HF experiments), only single-run single-batch experiments are run. This seems like the worst case for cost for the BO algorithms (incurring the batch overhead each time), so does not undermine any of the points or conclusions of the paper. However, it might be worth mentioning why this was done. Is that because the BO algorithms were not set up to be able to consider batch/multiple-run tradeoffs (i.e., because conventional available BO algorithms do not do this)? Another opportunity for future research?

D. Appropriate use of statistics and treatment of uncertainties

The paper is well-done from a statistical perspective: the paper shows repeated instances for the BO cases, and shows both the individual run and median costs associated with each optimization trajectory. Because the number of junior, senior, and lay humans is very small (only three in each case), it would help as mentioned above to show additional plots like Fig. S3 for these cases.

E. Conclusions: robustness, validity, reliability

Recommendations to the data and experiments have made in this review above, to better document or justify several of the statements and conclusions made in the paper.

F. Suggested improvements: experiments, data for possible revision

See earlier comments.

G. References

The references are appropriate, though some mention of existing Bayesian optimization approaches applied to semiconductor processes should be added to avoid an implication that this paper is the first to do so. E.g., C. Lang et al., “Modeling and Optimizing the Impact of Process and Equipment Parameters in Sputtering Deposition Systems Using a Gaussian Process Machine Learning Framework,” IEEE Trans. Semi. Manuf., 2021; Z. Chn et al., “A hierarchical expected improvement method for Bayesian optimization,” arxiv, 2021; S. Guler et al., “Bayesian optimization for Tuning Lithography Processes,” IFAC 2021.

H. Clarity and context

The paper is clear and well written, with good summary, introduction and context, and discussion of conclusions. Improvements in the methodology and discussion as recommended above should flow into abstract and conclusion sections as well. Other minor comments and suggestions follow. For Fig. 2, the three senior and three junior engineer trajectories should be shown each with unique line plotting characteristics, to better distinguish between them and not just between senior and junior. Throughout, “cummulative” should be “cumulative”.

Referee #2 (Remarks to the Author):

The paper is well organized one, and the idea is accepted. It will be appreciated to provide supplemental explanation based on below attached comments, for final publication -
1. In fact, the optimization process for the black-box model is an exploration-exploitation trade-off. The V-shape dependence of cost-to-target on amount of expert data is very crucial to make such human-AI collaboration work. Maybe the V-shape doesn't even exist in some worst cases. How to effectively benefit from it and what kinds of situations will fail can be discussed further. In addition, the transfer points ‘A’ to ‘E’ are used to evaluate the cost-to-target benchmark for the proposed Human First-Computer Last (HF-CL). Are there any criteria for picking these points? In the most industrial applications, it’s difficult to set up various transfer points for trials so that how to decide a good transfer point efficiently, e.g., a clear demarcation between rough tuning and fine-tuning? How to find the right switching point between the rough tuning and fine-tuning stages (how to define C point objectively) was not investigated in the paper.

2. Although the Bayesian optimization applied in this study is good a sequential design strategy for optimizing black-box functions, it is sensitive to cold-start settings and hyper-parameters including the selection of kernel function, search space, surrogate function. The table 1 in this paper shows that the three diverse varieties of Bayesian optimizations get different performance under the same transfer point, and the Gaussian processing significantly outperforms all other approaches in this study. The impacts of above issues could be further discussed from an algorithmic perspective. More analysis on explaining the reasons for Gaussian Processing method being the best method compared with the others such as the Markov Chain Monte Carlo (MCMC) method. This finding seems contradictory to what the author mentioned about MCMC being rated as one of the top ten most influential algorithms by IEEE. Also lacking of the comparisons with other non-Bayesian optimization algorithms.

3. This study is based on simulations on a virtual environment for a fair benchmark. However, in the semiconductor industry, the data may have noise, the equipment may be diversity under different processes. Besides, the Bayesian optimization is also weak at performing in high dimensionality. The generalization ability of the proposed method could be discussed. Finally, the exploration and exploitation are crucial to such optimization approaches. The exploration from AI sometimes might be go against the intuition/experience of experts. How to deal with this conflict?

Author Rebuttals to Initial Comments:

Referee 1:

The paper argues that combined human expert and AI algorithms have benefits for design of complex semiconductor process recipes, compared to a “from scratch” algorithm alone, or human expert alone. The methodology of the paper is to perform and analyze results from a challenge problem – to design a plasma etch process (find process recipe settings for 11 process parameters) that achieves within-specification results across six process performance metrics. Virtual fabrication process runs, with associated per-run and per-batch fabrication costs, are available to the designer (human and/or algorithm) in order to explore the design space and corresponding results. The goal for each designer is to meet specifications with as low a total fabrication cost as possible. The experimental results presented demonstrate substantially better (about 50% lower) costs for the combined “human first – computer last” (HF-CL) approach, than for the best performing experienced senior engineer expert alone.

Author Response:

The referee provides an excellent summary that shows understanding of our paper.

Referee 1:

The results are interesting, original, and relevant to the semiconductor technology community and beyond. The paper will be a valuable contribution to the field if key additional information, data, and issues can be addressed in revision of the paper.

Author Response:

Thank you for this comment.

Referee 1:

Several important areas or issues related to the methodology need additional information, for the reader to understand and be convinced by the baselines and comparisons presented. First, more clarity is needed about what information and methods the human designers had access to, for their design explorations. In Fig. 1, cross sections of etch features are shown; did the human experts have access to these plots, or only the values for the six-output metrics? In other words, is exactly the same information provided to both the humans and the algorithms?

Author Response:

We have added the suggested content to the manuscript. Humans and algorithms were provided the same information for each submitted recipe; however, the algorithms effectively ignored them. We edited two locations in the paper. The first edit is on page 4:

> As in the laboratory, the goal of the game is to minimize cost-to-target of finding a recipe that produces output metrics that meet the target. The participant submits a batch of experiments (one or more recipes) and receives output metrics and cross-sectional profile images. The participant continues to submit batches of recipes until the target is met, corresponding to the profile shown in Figure 1. We define a "trajectory" as a series of recipe batches carried out to meet the target.

The second is on page 6, to let the reader know that computers ignored the images:

> The algorithms proposed one recipe per batch by default[33] based on the output metrics, while effectively ignoring the output profile images. Trajectories were repeated 100 times for statistical relevancy to account for inherent randomness in cost-to-target due to the probabilistic nature of Bayesian optimization. To save computational time, trajectories were truncated if not meeting

---

Referee 1:

More importantly, are there restrictions on methods the humans could use (or, conversely, more description of the methods that the humans did use, should be included)? The paper refers to "experience and intuition," and to (presumably conventional engineering) training to use single or two-parameter at-a-time exploration. But well-trained engineers will typically employ conventional design of experiment (DoE) strategies, usually in conjunction with basic response surface modeling, regression, and statistical tools. Were the engineers allowed to use these basic engineering methods? Did they? Or were they restricted to just selecting conditions and observing outputs, without pencil, paper, spreadsheet, statistical package, or other tools? Or (hopefully and more persuasively) if they did have access to such methods, a description of what conventional tools they used can be included in the paper, so that the reader would understand that engineers struggle, even when using readily available and conventional engineering methods, in this kind of complex design problem.

Author Response:

We have added more information on the human engineer method to the manuscript. The engineers designed their experiments using mechanistic hypotheses based on prior knowledge of process trends and parameter dependencies. In contrast, statistical software would have required batches upwards of 25 to analyze the 11 input parameters. Process engineers generally use statistical software only in cases where they feel they can afford the large batch size, such as on test wafers or on very large datasets. The added text is on page 5:

> professional process engineers with PhD degrees in the physical sciences: three senior engineers with over five years of experience and three junior engineers with less than one year of experience (see Table S1). The engineers designed their experiments using mechanistic hypotheses based on their prior knowledge of process trends and plasma parameter dependencies. They chose an [illegible]

Since the reviewer correctly points out that human engineers use single or two-parameter at-a-time parameter changes, we provided statistics on how often this was used on page 5:

experiments, using univariate or bivariate parameter changes in 95% of their recipe choices. For reference, three inexperienced individuals with no relevant process experience also participated.

Referee 1:

Second, and in a related fashion, some additional discussion about the Bayesian optimization baselines is needed. The paper is clear that, for algorithm-only design trajectories, each start with 32 Latin hypercube experiments (and provides the range for parameters based on equipment limits). Bayesian optimization (BO) methods, however, also explicitly have some formulation of a prior belief, and it would help to mention or disclose those. Perhaps these are "non-informative" priors? Such limited prior belief approach is appropriate for the paper; however, the authors should note that this might be the most "difficult" starting scenarios for such algorithms. In practice, there is great interest in the field in imprinting some domain knowledge in the form of a good starting or prior belief (e.g., V. Fortuin, "Priors in Bayesian Deep Learning: A Review," arXiv, May 2021). Indeed, creating or learning a good prior might be considered competition to the HF-CL approach, or certainly is an area/approach worth mentioning as future research.

Author Response:

Thank you for pointing this out. We have added information on the prior used to the manuscript. The algorithms used non-informative priors. We clarified and added Fortuin as reference #34 on page 6:

bound (LCB) acquisition function. The algorithms started without any training and using non-informative priors.[32]

As suggested, we also added "imprinting domain knowledge in the form of a prior belief" as competition to the HF-CL strategy studied here and as an item of interest for future research on page 10:

domains may be harnessed to accelerate learning in novel domains.[41–43] Another area of interest in the AI field is imprinting domain knowledge in the form of a prior belief.[33,44] Indeed, creating or learning a good prior might be considered competition to the HF-CL strategy studied here. Other potential approaches in literature include incorporation of mechanistic physics models.[13]

Referee 1:

The methodology for HF-CL hybrid optimization needs some clarification. For the different "transfer" points from A to E, what information and exactly when is that information transferred to the BO algorithm? It is clear from the tables which experimental run data is transferred, but it is not clear when the narrower "Expert constraints" (**new Table S3**) are transferred to the BO algorithm. Is that starting at point A? Do they change or get tighter from A to B to E?

Author Response:

We have added the suggested content to the manuscript. The constrained search range was the same for all transfer points. It was transferred at point A and did not change. We clarified this in two different locations. The first location is on page 7:

> navigation. Therefore, we decided to test a hybrid strategy where the expert guides the algorithms in a Human First - Computer Last (HF-CL) scenario. In this implementation, the expert provides experimental data collected up to a transfer point labeled A through E in Figure 2, along with a constrained search range (Table S3). Finding the target with a random search

The second location is in the caption of Figure 3 on page 8:

> algorithms: (**a**) MCMC-EI (**b**) TPE-EI and (**c**) GP-LCB. The 'no human' results are for the algorithm without any help from humans, for reference only. Panels A through E show HF-CL results using the constrained range (Table S3) plus data from the expert up to that transfer point (inset of Fig. 2). Cost-to-target is a sum of the cost of data from the expert plus the cost of data

---

Referee 1:

This clarification about runs vs. constraints is especially important, because some statements are made in the paper that need better experimental explanation, or additional experiments, to better understand. In some places, the paper suggests it is too large an exploration space that challenges the BO algorithms (e.g., "the computational algorithms only became competent after the search space was simplified"), and elsewhere it is the lack of data ("little data") that limits the algorithms. Because BOTH additional experimental runs AND restricted parameter ranges are provided in A through E cases, it is hard to decouple these effects. What would have happened in the following cases: (1) only the restricted expert-provided parameter ranges were provided to BO; (2) only the specific runs but no change to the exploration space are provided to BO; vs. (3) providing both, as was done in the paper. Adding cases 1 and 2 to the experimental dataset would help decouple these issues and provide better justification for statements in the paper.

Author Response:

Thank you for pointing this out. We think this is an excellent suggestion. To separate the effects of providing both data and constraints, we added a new experiment for HF-CL *with* and *without* expert constraints (same expert data). We refer to new SI results on page 7:

> Figures S3 and S5 for results on other humans and Figure S4 for the expert *without* the constrained range. Once the computer takes over decision-making, the expert effectively

Figure S4 shows impressive cost-savings either way, but better performance *with* expert constraints. Thus, the engineer should preferably provide *both* data and a constrained range when implementing HF-CL in the laboratory. Here is Figure S4 and caption on page 25:

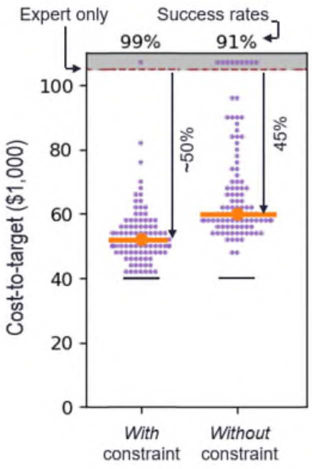

Fig. S4. Cost-to-target for HF-CL strategy *with* and *without* constraints. In this HF-CL implementation, the expert transfers data to the computer (Algo3) up to transfer point C either *with* or *without* a constrained search range. (An adaptive range parameter searching 20% beyond the data distribution is used *without* the constraint.) Cost-to-target is sum of cost from both the human and computer; each dot represents 100 independent trajectories; black vertical lines are cost transferred by the expert. Both represent significant cost savings relative to the expert alone: a median of 50% savings *with* the constrained range versus 45% savings *without* the constrained range. Improved performance *with* the expert constrained range suggests that the engineer should provide data *and* a constrained range when implementing HF-CL in the laboratory.

Referee 1:

The paper has an extended discussion of the V-shaped cost vs. amount of run information curves, for the combined HF-CL approach. It is stated in the paper that “the vertex for all algorithms in Fig. 3 corresponds to the inflection to the fine-tuning stage.” This might be true for the case shown in Fig. 3, for the one succeeding senior engineer. But this does not seem to be the case for the one succeeding junior engineer, as shown in Fig. S3, where point A (quite far from “fine tuning”) is very nearly the same as point B, in cumulative cost. So it may be that the paper discussion of the V shape is too narrowly dependent on the “best expert” experiments that were run. Indeed, a good (junior) domain expert combined with the BO GP algorithm (**now called Algo3**) seemed to achieve impressive benefits, just by getting the algorithm started even with perhaps $20-30K of experiments.

Author Response:

Thank you for pointing this out. We modified to clarify that the overlap of the inverted regime with the fine-tuning stage suggests this stage is better relegated to computer algorithms (versus claiming the vertex is *exactly* at the inflection to the fine-tuning stage). We rephrased on page 9:

intuition even for a highly experienced engineer has significantly diminished, enabling the computer algorithms to become statistically more competent at choosing recipes. The overlap of the inverted regime with the fine-tuning stage suggests this stage may be better relegated to computer algorithms – such as Bayesian optimization algorithms. The observation of the V-shaped phenomenon for different human and computer combinations strengthens our belief that

To give more perspective on the "impressive" benefits for the computer partnered with the junior engineer, we also note that the *absolute* cost-to-target is still relatively high because the junior engineer started with a higher cost-to-target than the senior engineer. We modified the caption of Figure S3 on page 24:

the median cost of HF-CL at the transfer points using Algo3, showing a V-shaped dependence of the cost-to-target on the amount of data transferred from the human to the computer. HF-CL provides impressive cost-savings when partnered with the junior engineer at point A' or B'. Yet note that the *absolute* cost-to-target is still comparably higher than when partnered with the expert (for reference, the same algorithm partnered with the expert achieved a median cost-to-target of $52,000).

Referee 1:

Related to Fig. S3, it is not clear if the junior engineer also provided narrower parameter exploration ranges to the BO algorithm? If so, those should be added as a column in Table S2 (new Table S3).

Author Response:

We have added the suggested content to the manuscript. The junior engineer provided the same *size* exploration range as the senior engineer. Ranges are different because the junior engineer explored a different regime. To clarify in the text, we added a column to Table S3 (and more for other engineers) and explained in the caption on page 31:

Input parameters	Unconstrained	SE1	SE2	SE3	JE1	JE2	JE3
Pressure (mT)	5 – 120	12 – 30	12 – 30	5 – 23	5 – 23	20 – 38	12 – 30
Power 1 (W)	0 – 29,000	4,000 –15,000	4,000 –15,000	4,000 –15,000	4,000 – 15,000	14,000 – 25,000	4,000 – 15,000
Power 2 (W)	0 – 10,000	1,000 – 7,000	1,000 – 7,000	0 – 6,000	1,000 – 7,000	2,000 – 8,000	0 – 6,000
Ar flow (sccm)	0 – 1,000	100 – 400	0 – 300	0 – 300	0 – 300	300 – 600	0 – 300
C_4F_8 Flow (sccm)	0 – 100	20 – 60	20 – 60	10 – 50	0 – 40	40 – 80	20 – 60
C_4F_6 Flow (sccm)	0 – 100	22 – 66	15 – 59	10 – 54	0 – 44	0 – 44	12 – 56
CH_3F Flow (sccm)	0 – 20	0 – 5	0 – 5	0 – 5	7.5 – 12.5	3 – 8	15 – 20
O_2 Flow (sccm)	0 – 50	20 – 50	20 – 50	10 – 40	0 – 30	10 – 40	20 – 50
Pulse duty cycle (%)	10 – 100	20 – 60	30 – 70	10 – 50	10 – 50	10 – 50	10 – 50
Pulse frequency (Hz)	500 – 2000	1000	1000	1000	1000	1000	1000
Temperature (°C)	-15 – 80	20 – 45	10 – 35	30 – 55	20 – 45	25 – 50	15 – 40

Table S3. Input parameter search ranges. Unconstrained range and constrained ranges given by the professional engineers in the HF-CL strategy. SE= senior engineer and JE=junior engineer. The constraints reduce each parameter by roughly one-quarter to one-half of the original range. All constrained ranges are the same size to simplify comparison; ranges differ as humans explored different regimes. ("sccm" = standard cubic centimeter per minute)

Referee 1:

In terms of data and methodology, having Fig. S3 is a great help – i.e., seeing the HF-CL median cost for the (succeeding) junior engineer helps contrast with the curve for the (succeeding) senior engineer. It would also be informative to show similar plots for the other two senior engineers and other two junior engineers; even though they did not succeed on their own in meeting specifications, it would bolster or shed light on the argument about HF-CL being a good approach, to elucidate if the approach works also

in conjunction with other human designers (and not just in combination with engineers who were able to meet the specifications on their own). Indeed, it might even be interesting to see a similar "V" curve (or lack thereof) for the lay-person cases – to better make the case that domain expertise helps or is needed as part of the HF-CL approach and buttress the claim that "domain knowledge remains indispensable in navigating the earlier stages of process development."… Because the number of junior, senior, and lay humans is very small (only three in each case), it would help as mentioned above to show additional plots like Fig. S3 for these cases.

Author Response:

This is an excellent suggestion. In response, we added an additional plot to SI for analysis of all engineers plus an inexperienced participant, to further support our claim that domain knowledge is indispensable to the HF-CL approach. Figure S5 compares cost-to-target using the HF-CL strategy for an equivalent amount of data transferred to the computer. The results support that HF-CL strategy is more effective at lowering costs when partnered with more experienced humans. Here is Figure S5 and caption on page 26:

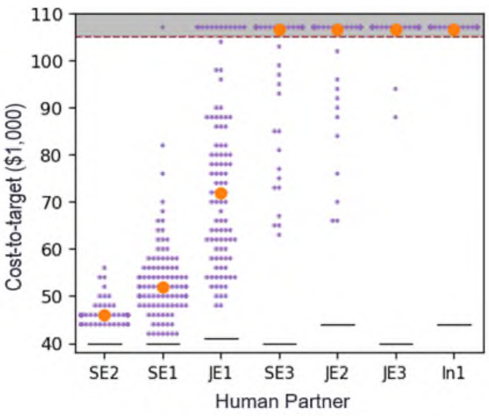

Fig. S5. HF-CL strategy using different human participants. Results for different humans partnered with Algo3 in the HF-CL strategy. SE = senior engineer; JE= junior engineer; In= inexperienced player. See Table S1. (Note that SE1 is point C in Figure 3c; JE3 is point C' in Figure S3.) Each human transferred an equivalent of $40,000 of data (or nearest full batch, see Table S3) along with a constrained search range (Table S7) to the computer. Since In1 did not have enough experience to constrain the range, an adaptive range parameter searching 20% beyond the data distribution was used. Cost-to-target is the sum of cost from both the human and computer; each dot represents 100 independent trajectories; orange lines are median cost-to-target; black vertical lines indicate cost transferred from the human. The results are plotted from left to right by increasing *absolute* cost-to-target (as not all humans met target). Lowest costs are associated with the highest experience levels. Overall, the results support that HF-CL strategy is more effective at lowering costs when partnered with more experienced humans.

We also added a sentence to the abstract on page 2:

the advantages of human experts and algorithms into a "Human First-Computer Last" (HF-CL) strategy can reduce cost-to-target by half relative to the human alone. The HF-CL strategy is more effective at lowering costs when partnered with more experienced humans. Even with an expert, the HF-CL success still depends on *when* the computer algorithm is deployed, with a V-

---

Referee 1:

A small side comment is also worth mentioning, or suggesting, for Table S2 (**new Table S3**). It is hard for the reader to get a sense of how big the solution (not the exploration) space is for this design problem. In other words, maybe there are many combinations and ranges of process parameters that achieve acceptable results? Or is there a relatively small space, in the end, where the process recipe has to sit, in order to meet specifications? One way to convey this might be to add another column to Table S2 (**new Table S3),** that shows, across all successful recipes, what the ranges in each of the 11 process recipe parameters were (e.g., perhaps we find that O2 flow ALWAYS had to be in the 22 to 23 sccm range for a recipe to work.)

Author Response:

This is a good question. We have added calculations to the manuscript to help the reader get a sense of the solution space. We provide an estimate of the random chance of meeting target in the *unconstrained* range on page 4 and 6:

> Estimated from actual costs, we assign a cost of $1,000 per recipe for wafer and metrology costs, and an overhead cost of $1,000 per batch for tool operation. We verified at the outset low odds of randomly meeting target: 0.003% per recipe based on 35,000 random samples.

> $105,000). We define "success rate" as the percentage of trajectories that meet target at a lower cost-to-target than the expert benchmark. For reference, the success rate by pure chance alone is estimated to be only 0.3% (based on 0.003% odds per recipe mentioned earlier).

We also added similar estimates in the *constrained* range on page 7:

> algorithms in a Human First - Computer Last (HF-CL) scenario. To implement this strategy, the expert provides experimental data collected up to a transfer point labeled A through E in Figure 2, along with a constrained search range (Table S3). The constrained range is about a hundred-times more promising than the original range, with a 23% success rate for a random search (based on a 0.27% per recipe chance of randomly meeting target on 2,700 random samples). See Figures S3-S5 for HF-CL results *without* the constrained range and on other humans. Once the computer takes over decision-making, the expert effectively relinquishes control and has no

As for winning recipes, there is no special parameter range because changing one parameter can be compensated for by changing another due to high degeneracy of the input parameter space. We added this information on page 4:

> Estimated from actual costs, we assign a cost of $1,000 per recipe for wafer and metrology costs, and an overhead cost of $1,000 per batch for tool operation. Many potential winning recipes exist due to high levels of degeneracy in the input parameter space. Yet, we verified at the outset low odds of randomly meeting target: 0.003% per recipe based on 35,000 random samples.

Referee 1:

Fig. S5 (**now Figure S7**) shows three different examples of HF-CL trajectories, in support of the claim that the BO algorithmic trajectories are often quite different than the progressively improving human expert trajectories. However, this plot is shown for the TPE algorithm (**now called Algo2**), which was the

worst performing of the three BO approaches in Fig. 3. It would be better to show sample trajectories for the BO GP algorithm (**now called Algo3**), since that was presented as the best or winning approach (and avoids the reader wondering if the trajectories of Fig. S5 (**now Figure S7**) are due to, and a manifestation of, it being an inferior algorithm in this case).

Author Response:

We have added the suggested content to the manuscript by adding trajectories for all algorithms in Figure S7 on page 28:

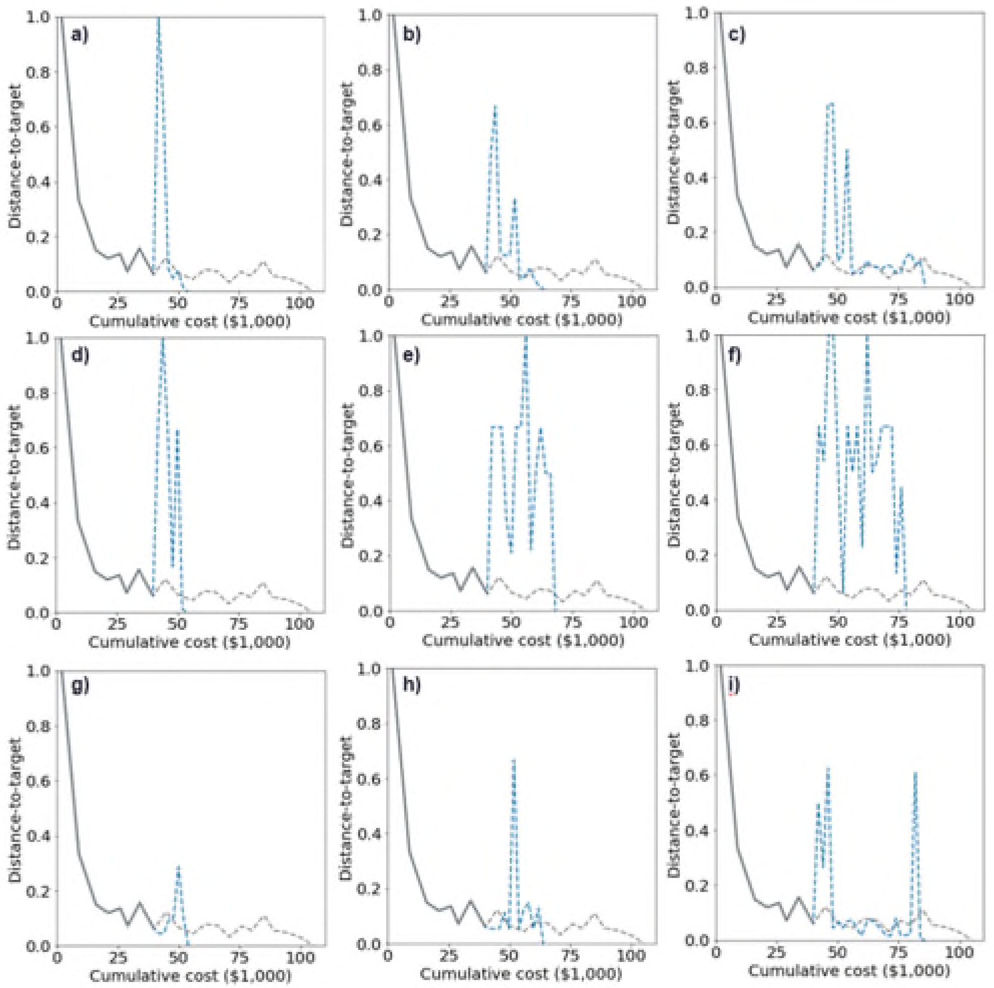

Fig. S5. Sample trajectories for the HF-CL approach. The expert trajectory shown in grey, with transfer to the computer at $40,000 (point 'C' in Figure 2). The blue line is the trajectory of the algorithm; the dotted grey line is the continuation of the trajectory for the expert only. Distance-to-target is plotted as a rolling minimum of one batch (no smoothing of the curve). The algorithm used in panel **a, b, c** is MCMC-EI, in **d, e, f** is TPE-EI, and in **g,h,i** is GP-LCB.

Referee 1:

For the BO optimization, after the first 32 runs (as well as after the transfer from HF experiments), only single-run single-batch experiments are run. This seems like the worst case for cost for the BO algorithms (incurring the batch overhead each time), so does not undermine any of the points or conclusions of the paper. However, it might be worth mentioning why this was done. Is that because the BO algorithms were not set up to be able to consider batch/multiple-run tradeoffs (i.e., because conventional available BO algorithms do not do this)? Another opportunity for future research?

Author Response:

This is a good point. We have added an explanation of the BO batch size to the manuscript. BO is designed by default to suggest one recipe (See Ref 33 Section 3.3). We added this detail and a citation on page 6:

> In their process games, the algorithms requested one recipe per batch (default for Bayesian optimizations)[33] and used only the output metrics, effectively ignoring the output profile images. Trajectories were repeated 100 times for statistical relevancy to account for inherent randomness

We are aware of more complicated BO strategies that make multiple proposals (Ref 25 "constant Liar" strategy, for example). Thus, we list 'batch size' as an opportunity for future research on page 10:

> exploration space, the computer may rely even more on domain knowledge, in effect delaying transfer to the computer. Other important factors could include process variability (noise), target tolerance, batch size, and cost structure. We have much to learn. These topics are good candidates for further systematic study on the virtual process platform.

We also point out the difference in batch size for human versus computer on page 10:

> while the computers employed multivariate parameter changes. Humans may find it difficult to accept recipes that they do not understand. (2) The engineers requested an average of four experiments per batch, while the computers requested only one experiment per batch – likely to be viewed as inefficient in the laboratory. (3) Process engineers steadily progressed towards target (Figure 2), while the computers utilized exploratory recipe-choice strategies that appear

---

Referee 1:

The paper is well-done from a statistical perspective: the paper shows repeated instances for the BO cases and shows both the individual run and median costs associated with each optimization trajectory. Because the number of junior, senior, and lay humans is very small (only three in each case), it would help as mentioned above to show additional plots like Fig. S3 for these cases.

Author Response:

Thank you. As mentioned above, we added more analysis on other humans to Table S5 and direct acknowledgment of the small number of humans on page 9:

> shaped phenomenon for different human and computer combinations strengthens our belief that our insights are generalizable to this *little* data problem, despite the relatively small number of humans. Furthermore, we believe the V-curve phenomenon is a natural consequence of trying to minimize cost *in the limit of* expensive data and tight tolerances – as is the case in many

---

Referee 1:

The references are appropriate, though some mention of existing Bayesian optimization approaches applied to semiconductor processes should be added to avoid an implication that this paper is the first to do so: Lang et al., "Modeling and Optimizing the Impact of Process and Equipment Parameters in Sputtering Deposition Systems Using a Gaussian Process Machine Learning Framework," IEEE Trans. Semi. Manuf., 2021; Chen et al., "A hierarchical expected improvement method for Bayesian optimization," arxiv, 2021; S. Guler et al., "Bayesian optimization for Tuning Lithography Processes," IFAC 2021.

Author Response:

Per the suggestions of the reviewer, we have added references #23, #24, and #25.

Referee 1:

The paper is clear and well written, with good summary, introduction and context, and discussion of conclusions. Improvements in the methodology and discussion as recommended above should flow into abstract and conclusion sections as well. Other minor comments and suggestions follow. For Fig. 2, the three senior and three junior engineer trajectories should be shown each with unique line plotting characteristics, to better distinguish between them and not just between senior and junior. Throughout, "cummulative" should be "cumulative".

Tracking# 2022-01-00365A

Author Response:

We corrected "cumulative." In Figure 2, we re-colored trajectories with unique colors in figure 2 on page 5:

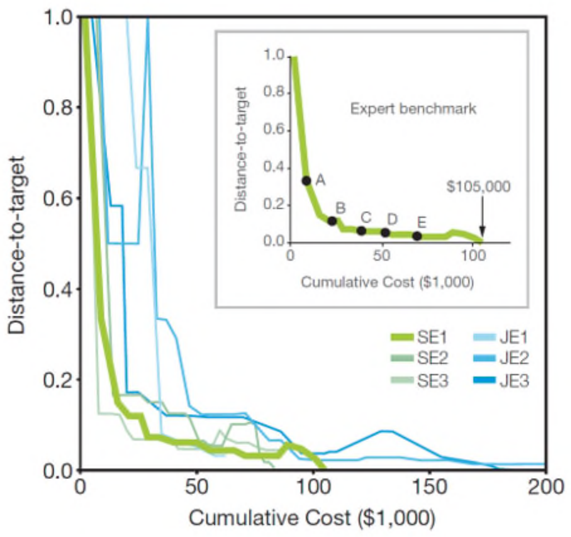

Fig. 2. Trajectories of the human engineers. Results for senior engineers in green and junior engineers in blue defined in Table S1. Results for inexperienced participants are in Figure S2. The calculation for distance-to-target is explained in SI. Inset is the "expert" (SE1) trajectory showing transfer points A through E used in the HF-CL strategy.

Referee 2:

The paper is well organized one, and the idea is accepted. It will be appreciated to provide supplemental explanation based on below attached comments, for final publication.

Author Response:

Thank you for this comment.

Referee 2:

In fact, the optimization process for the black-box model is an exploration-exploitation trade-off. The V-shape dependence of cost-to-target on amount of expert data is very crucial to make such human-AI collaboration work. Maybe the V-shape does not even exist in some worst cases. How to effectively benefit from it and what kinds of situations will fail can be discussed further.

Author Response:

We have added the suggested content to the manuscript by clarifying two examples when the V-curve might not exist: (1) relaxed constraints and (2) chamber matching on page 9:

> opment. We showed that the position and depth of the vertex depends on the specific algorithm and human. In addition, we expect the right side of the V might not exist if targets were relaxed or, conversely, might dominate in processes that only need re-tuning such as in chamber matching (i.e., transferring a known process to a similar tool). Other important factors

Referee 2:

In addition, the transfer points 'A' to 'E' are used to evaluate the cost-to-target benchmark for the proposed Human First-Computer Last (HF-CL). Are there any criteria for picking these points?

Author Response:

Our primary criterium for picking a transfer point is that full batches of recipes are included, so as not to separate any recipes in the batches. For the transfer point comparing different humans, we used the expert point C as baseline and then used equivalent costs for the other humans, or as close as possible to complete a batch. The transfer points are defined in Tables S5, S6, and S7 on pages 33-35.

Transfer point	A	B	C	D	E
Recipes transferred	7	17	32	41	53
Batches transferred	2	4	8	10	13
Cost transferred	$9,000	$21,000	$40,000	$51,000	$66,000

Transfer point	A’	B’	C’	D’	E’
Recipes transferred	11	19	27	47	71
Batches transferred	2	6	14	12	15
Cost transferred	$13,000	$29,000	$40,000	$59,000	$86,000

Transfer point	SE1	SE2	SE3	JE1	JE2	JE3	In1
Recipes transferred	32	28	27	35	27	32	35
Batches transferred	8	12	14	9	13	8	9
Cost transferred	$40,000	$40,000	$41,000	$44,000	$40,000	$40,000	$44,000

Referee 2:

In the most industrial applications, it is difficult to set up various transfer points for trials so that how to decide a good transfer point efficiently, e.g., a clear demarcation between rough tuning and fine-tuning? How to find the right switching point between the rough tuning and fine-tuning stages (how to define C point objectively) was not investigated in the paper.

Author Response:

We agree with the reviewer’s assessment. Accordingly, we acknowledge this challenge and added text on page 9:

> For the industry to implement the lessons of the HF-CL approach to actual semiconductor processes, it will be essential to understand how the insights apply to other processes and when humans should give up control – namely, how to identify the ideal transfer point during development. The lateral position and depth (i.e., maximum cost savings) of the vertex will

---

Referee 2:

Although the Bayesian optimization applied in this study is a good sequential design strategy for optimizing black-box functions, it is sensitive to cold-start settings and hyper-parameters including the selection of kernel function, search space, surrogate function. Table 1 in this paper shows that the three diverse varieties of Bayesian optimizations get different performance under the same transfer point, and

the Gaussian processing significantly outperforms all other approaches in this study. The impacts of above issues could be further discussed from an algorithmic perspective. More analysis on explaining the reasons for Gaussian Processing method being the best method compared with the others such as the Markov Chain Monte Carlo (MCMC) method (**now called Algo1**). This finding seems contradictory to what the author mentioned about MCMC being rated as one of the top ten most influential algorithms by IEEE. Also lacking the comparisons with other non-Bayesian optimization algorithms.

Author Response:

Thank you for pointing this out. To address the reviewer's suggestion, throughout the manuscript we renamed the BO algorithms more simply and added a table to clarify the details of each algorithm. The added Table S2 can be found on page 30:

	Algo1	Algo2	Algo3
**Sampling method to compute the posterior distribution**	*Markov Chain Monte Carlo*	Does not compute posterior because it uses classification	No sampling because closed form solution for posterior exists
**Surrogate model**	*Multivariate linear*	*Tree-Parzen Estimator (good/bad classifier)*	*Gaussian process*
**Acquisition function**	Expected Improvement	Expected Improvement	*Lower Confidence Bound*
**Objective function**	Scaled Euclidean distance	Scaled Euclidean distance	Scaled Euclidean distance
**Recipes per batch (after initial seed)**	1	1	1
**Priors**	Non-informative	Non-informative	Non-informative

Then, we also clarified the BO algorithms in the main text on page 6:

> diverse varieties of Bayesian optimizations were selected (see Table S2): (1) Algo1 using MCMC-EI with Markov Chain Monte Carlo sampling[19,26,27] a multivariate linear surrogate model (to compensate for the high computation cost of this method), and an expected improvement (EI) function. (2) Algo2 from an open-source software using the Tree-structured Parzen Estimator with an EI acquisition function.[28–30] (3) Algo3 using a Gaussian Process model[31] and an acquisition function different from the others: lower confidence bound (LCB).

This allows us to explain our thinking for why Algo3 outperforms the others. Our added explanation can be found on page 7/8:

> adds cost without clear benefit to the algorithm. The optimal performance for all algorithms is at point C. Algo3 significantly outperforms the other algorithms, attributed to either the flexibility of Gaussian process models compared to simple linear or classification models or to its different acquisition function, as LCB has been shown to outperform EI.[33] This sets a new game benchmark with a (median) cost-to-target of $52,000 – at half the cost required by the expert

Finally, we added reference to Liang (Ref #33) and removed the IEEE reference.

Referee 2:

This study is based on simulations on a virtual environment for a fair benchmark. However, in the semiconductor industry, the data may have noise, the equipment may be diversity under different processes. Besides, the Bayesian optimization is also weak at performing in high dimensionality. The generalization ability of the proposed method could be discussed.

Author Response:

We agree with the reviewer that noise could be an important factor in the performance of the HF-CL strategy. Fortunately, BO inherently takes noise and variability into account probabilistically, treating data as a stochastic process (see Ref # 22, for example). Please see below where we already mention 'noise' in our list of possible future studies on page 10:

> delaying transfer to the computer. Other important factors could include process variability and noise, target tolerance, batch size, constrained range, and cost structure. We have much to learn. These topics are good candidates for further systematic study on the virtual process platform.

We also agree that the topic of higher dimensionality is also very interesting and relevant to process development. For this factor, we added another sentence to our discussion on page 10:

> disappear if targets were relaxed or, conversely, dominate in processes that only need re-tuning such as chamber matching (transferring a process to a similar tool). In a high dimensionality exploration space, the computer may rely more on domain knowledge, effectively delaying transfer to the computer. Other important factors could include process variability (noise), target [illegible]

Referee 2:

Finally, the exploration and exploitation are crucial to such optimization approaches. The exploration from AI sometimes might be go against the intuition/experience of experts. How to deal with this conflict?

Author Response:

We agree with the reviewer that there may be a conflict when the computer behaves differently from the human. This conflict is already highlighted in the paragraph on cultural hurdles on page 10:

> steadily progress towards target, as in the trajectories of Figure 2. Yet, we observed the computer algorithms utilizing exploratory recipe-choice strategies that could appear sacrificial (Fig. S6). Counter-intuitive moves by computer algorithms are well documented in game-playing, as programmed computers do not play emotionally.[41] In the real-world, process engineers will need to resist intervening in the algorithmic strategy and inadvertently raise cost-to-target, even when

Reviewer Reports on the First Revision:

Referee #1 (Remarks to the Author):

The revision has substantially improved the paper, and the authors are commended for their work to well-answer most of the previous concerns. Two substantial issues remain to be addressed – one critical, and one important but not critical. These are discussed in the “Data and methodology” review component below.

A. Summary of key results
The revision has substantially improved the presentation and results of the paper. One key result has been substantially clarified with an additional experiment, now showing the additional cost savings by adding expert parameter constraints on top of the provided human-defined experimental sample points at transfer point C.

B. Originality and significance
As noted in the previous review, the results are interesting, original, and relevant to the semiconductor technology community and beyond. Two remaining issues need to be clarified or addressed, to ensure that the results are significant and properly framed to support the conclusions.

C. Data and methodology
The first and most critical concern relates to the methodology employed in the three algorithms, particularly related to the framing of the “game” or optimization problem that these algorithms employ. What exactly is the optimization problem formulation? The paper highlights that the humans and the algorithms are seeking to minimize the experimental cost to find a recipe that brings all six outputs to within some specification (Fig. 1 and Table S4). However, the paper also shows trajectories and compares results using a “distance-to-target” calculation. Is this the objective function that is used by each or some of the algorithms?
If this distance-to-target metric is indeed how the “game” has been translated into the algorithms, I believe there is a potentially fatal flaw in the methodology: this particular distance-to-target metric used will artificially induce the algorithms to struggle in earlier stages of optimization, and only succeed in a fine-tuning regime. That is to say, this particular (and somewhat odd) distance-to-target metric may be responsible for the primary conclusions of the paper – that a human first and computer second approach is the most effective – but for an accidental or erroneous reason. The essential problem is that this particular distance-to-target metric only provides relative goodness when an output is “close to target.” When far away (either too large or too big), a score of 1 is assigned for that output metric. This is problematic in two respects: it does not allow the algorithm to know if the output is too big or too small, just that it’s “too far” away. Second, it does not allow comparison of two different sample point results that happen to both give far-away from target results; such relative comparison of goodness is essential to gradient calculation or consideration of best conditions to consider next. In essence, only when a target randomly, or when given human-provided samples, then happen to be within the “close” regime, does the output metric scale from 0 to 1; only in this region do the algorithms have the essential gradient/directionality information that enable them to drive the rest of the way to the target specification. Until that point (and indeed, for each different output), the algorithms are most likely just inefficiently and ineffectively further

random sampling.
Why specify and use such an odd and non-informative distance-to-target metric? Truncating successful or within-spec output metrics to 0 is justifiable; in this game, there is no relative advantage in further driving such metrics to a specific target. However, the truncating to 1 for “far away” results is not typical, or justified given the framing of the “game.” If the algorithms have been set up to optimize this particular distance-to-target rather than a more appropriate non-truncated (and gradient-providing) distance metric, then the algorithms have been handicapped in a way that the humans have not. And most critically, this odd distance-to-metric objective might thus lead to, or indeed, erroneously give rise to, the conclusions of the paper.
This concern may be due to lack of information in the paper about the specific objective functions being used by the algorithms (particularly the best performing Algorithm 3). If the distance-to-metric function described in the paper is just used to illustrate progress, but is not what is used by the algorithms, the above critical concern would be substantially reduced with clarification of algorithm setups.
A second (important but not blocking) issue has been half addressed by the revision, but the other half of the question remains. The issue is that both human-specified experiments and human-specified narrower constraints are provided at each transfer point. The added experiment (at transfer point C) shows that if constraints are provided in addition to the sample points, about 5% additional cost savings are achieved. But what if just constraints are provided to the algorithms? Maybe that is enough information that, with the dramatically reduced search space, a latin-hypercube sampling (LHS) starting from that information only might better use the available experimental points? (Note that shrinking each parameter space by ½, across 11 parameters, is over a 2000-fold search space reduction, so this could be quite important). Imagine a transfer point “L” (for human-recommended limits) to the left of transfer point “A”, but to the right of the “no human” point. One might be L+12 for improved limits and then 12 LHS points; another might be L+32 for algorithm given improved limits and then conducting 32 LHS samples within the smaller space. Note that the motivation for both L+12 and L+32 is that this “starting simulation sample size” is a challenging hyperparameter in surrogate modeling based methods (like BayesOpt), and considering both of these would also address some concern about neglecting this point in the paper. Note also that these are different from transfer point A, where human-provided sample points are also provided, but with the human-induced cost also incurred and the algorithm limited to the human-provided samples; it is possible that random sampling within the reduced space (and especially with more informative distance optimization objective as discussed above) would be better competition to the human sample points. More broadly, such evaluation would help answer the question about relative value of constraints, human-provided, and startup random sampling toward human-algorithm symbiotic improvements, and better disambiguate “human-provided information.”
Side note: the paper is not clear on when or if LHS sampling is done only for the “no human” case, or also in conjunction with transfer points A through E. My interpretation or assumption above, is that in transfers A through E, random sampling startup has been replaced by the human-provided samples. It would be good for the paper to make that explicit or clarify.

D. Appropriate use of statistics and treatment of uncertainties

Excellent additions by the authors, to provide valuable information about each human and trajectories or behaviors in those cases.

E. Conclusions: robustness, validity, reliability
I am concerned about the validity of the results, related to the particular “distance-to-metric” metric if it or substantially similar framings were used by the algorithms. The authors need to clarify and address this critical concern.

F. Suggested improvements: experiments, data for possible revision
See earlier comments.

G. References
Good additions by the authors.

H. Clarity and context
In two places, the writing seems to suggest that the algorithms choose to ignore information or make decisions, whereas the algorithms had no such choice or decision available to them. One relates to the use of the cross-sectional profiles; was there any kind of setup implemented where the algorithms could have used these profiles, but somehow elected not to and ignored them instead (line 148)? Similarly, at line 285 the paper says “while the computers requested only one experiment per batch.” But the algorithms didn’t get to choose: they were inherently set up or limited to only one experiment per batch, so it’s odd to use “request” wording to contrast with humans who were enabled to make the batch choice.
One more “cummulative” correction needed – Fig. S2 was corrected but Fig. S3 still has “cummulative” on the horizontal axis.
Labels for human participants in Table S1 appear confused (for JE1 and JE3). I hope that the company is looking to fast-track and promote junior engineer #1; they had impressive engineering performance in this game , both alone and when teamed with a computer algorithm.

Referee #2 (Remarks to the Author):

Thanks to the authors for the response. Most of the comments have been addressed. The revised manuscript is clearer for readers to understand how humans and AI can work together to accomplish a shared goal.

Author Rebuttals to First Revision:

Tracking# 2022-01-00365B

Referee 1:

The revision has substantially improved the paper, and the authors are commended for their work to well-answer most of the previous concerns. Two substantial issues remain to be addressed – one critical, and one important but not critical. These are discussed in the "Data and methodology" review component below.

Author Response:

We are glad we answered most of your concerns. Regarding the first issue, our responses below aim to clarify this metric was *not* used by any algorithms – it was *only* used to illustrate progress in the "game." To emphasize to other readers, we renamed this metric a "progress tracker." We thank you for motivating us to make this critical modification to our terminology. For the second issue, we added the requested data as discussed below.

Referee 1:

The revision has substantially improved the presentation and results of the paper. One key result has been substantially clarified with an additional experiment, now showing the additional cost savings by adding expert parameter constraints on top of the provided human-defined experimental sample points at transfer point C.

Author Response:

Thank you!

Referee 1:

As noted in the previous review, the results are interesting, original, and relevant to the semiconductor technology community and beyond. Two remaining issues need to be clarified or addressed, to ensure that the results are significant and properly framed to support the conclusions.

Author Response:

We provide a point-by-point response below.

Referee 1:

The first and most critical concern relates to the methodology employed in the three algorithms, particularly related to the framing of the "game" or optimization problem that these algorithms employ. What exactly is the optimization problem formulation? The paper highlights that the humans and the

algorithms are seeking to minimize the experimental cost to find a recipe that brings all six outputs to within some specification (Fig. 1 and Table S4).

Author Response:

Correct, the framing of this "game" is to minimize cost-to-target. We did not dictate any objective function to the participants.

Referee 1:

However, the paper also shows trajectories and compares results using a "distance-to-target" calculation. Is this the objective function that is used by each or some of the algorithms?

Author Response:

No, this metric was not the objective function used by the algorithms, instead they used a scaled Euclidian distance. We think the confusion is the word "distance." Therefore, to emphasize our metric is being used to track progress (and is not the objective function), we renamed it the "progress tracker" in all relevant figures and in the text. For example, here is Figure 2 re-labeled:

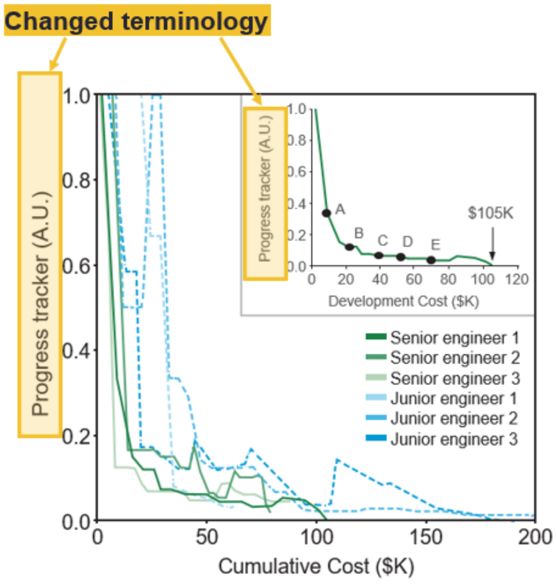

Referee 1:

If this distance-to-target metric is indeed how the "game" has been translated into the algorithms, I believe there is a potentially fatal flaw in the methodology: this particular distance-to-target metric used will artificially induce the algorithms to struggle in earlier stages of optimization, and only succeed in a

fine-tuning regime. That is to say, this particular (and somewhat odd) distance-to-target metric may be responsible for the primary conclusions of the paper – that a human first and computer second approach is the most effective – but for an accidental or erroneous reason. The essential problem is that this particular distance-to-target metric only provides relative goodness when an output is "close to target." When far away (either too large or too big), a score of 1 is assigned for that output metric. This is problematic in two respects: it does not allow the algorithm to know if the output is too big or too small, just that it's "too far" away. Second, it does not allow comparison of two different sample point results that happen to both give far-away from target results; such relative comparison of goodness is essential to gradient calculation or consideration of best conditions to consider next. In essence, only when a target randomly, or when given human-provided samples, then happen to be within the "close" regime, does the output metric scale from 0 to 1; only in this region do the algorithms have the essential gradient/directionality information that enable them to drive the rest of the way to the target specification. Until that point (and indeed, for each different output), the algorithms are most likely just inefficiently and ineffectively further random sampling.

Author Response:

Since the algorithms did not use this metric, there should be no concern that this metric affected their performance or influenced the conclusions of this paper. We hope our clarification resolves this issue.

It might help if we explain how we designed the "progress tracker" based on our experience in the lab. Engineers typically show customers progress using a "control table" where process outputs (such as etch rate, CD, etc) are color-coded depending on whether they met, are close to, or have failed to reach target. There is no standard metric to represent this table, so we designed the "progress tracker" for this purpose. Our progress tracker is an indicator from 0 to 1 for whether process met target (score=0), fails (score=1), or is close to target (scored between 0-1). We classify etch stop and mask consumption as failures (score=1). This way of defining the "progress tracker" helps us visually show progress with several advantages: it defines 0 as meeting target, so viewer can easily "see" when target is met. Also, the upper-bound of 1 allows easier viewing of multiple trajectories on the same plot. Lastly, it treats special cases as "far" from target (score=1). For example, top CD and mask remaining still meet target even with the process etch stops. Overall, we consider the "progress tracker" an effective way to visually monitor progress towards target in this "game." We added more information to SI on page 21:

Added explanation and clarification in SI

Calculation for "Progress tracker"

The "progress tracker" is our metric for monitoring how close a process is to target. To clarify, this metric is only to illustrate progress, it was not shown to any participants or used by any computer algorithms. In practice, process engineers monitor progress to target using a "control table" where process outputs (such as etch rate, CD, etc) are color-coding depending on whether they met, close to, or failed to reach target. There is no standard single-value metric to represent this entire table, so we designed the "progress tracker" for this purpose. Our progress tracker is an indicator from 0 to 1 for whether process met spec (=0), fails (=1), or is somewhere in between (0-1). We classify etch stop and mask consumption as failures (=1).

To calculate the "progress tracker" we take the mean of six scores from the six-output metrics, normalized to 1, using the definitions in Table S4. Each output metric is assigned a score of 0 if it meets the target values. (All metrics must have a score of 0 for the process to meet target.) An output metric is assigned a score of 1 if it is *far from* target. For output metrics that are *close to* target, the score was decreased linearly from 1 to 0. The progress tracker is also assigned as 1 if the process fails due to etch stop (etch depth less than 2000 nm) or if no mask remains (mask remain equals 0). Once progress tracker values are computed for every experiment, the progress tracker is then plotted as the best score per batch with a rolling window of four batches in Figures 2 and S2, and one batch in Figure S7.

Referee 1:

Why specify and use such an odd and non-informative distance-to-target metric? Truncating successful or within-spec output metrics to 0 is justifiable; in this game, there is no relative advantage in further driving such metrics to a specific target. However, the truncating to 1 for "far away" results is not typical, or justified given the framing of the "game." If the algorithms have been set up to optimize this particular distance-to-target rather than a more appropriate non-truncated (and gradient-providing) distance metric, then the algorithms have been handicapped in a way that the humans have not. And most critically, this odd distance-to-metric objective might thus lead to, or indeed, erroneously give rise to, the conclusions of the paper.

Author Response:

Please see explanation above for how we designed this "progress tracker." We believe it reflects how engineers and their customers "see" process results. The Euclidean distance used by the algorithms can vary from 0 to infinity, and thus does not truncate "far" from target.

Referee 1:

This concern may be due to lack of information in the paper about the specific objective functions being used by the algorithms (particularly the best performing Algorithm 3).

Author Response:

We previously reported the objective functions in SI. See highlighted row in Table S2:

	Algo1	Algo2	Algo3
Sampling method to compute the posterior distribution	Markov Chain Monte Carlo	Does not compute posterior because it uses classification	No sampling because closed form solution for posterior exists
Surrogate model	Multivariate linear	Tree-Parzen Estimator (good/bad classifier)	Gaussian process
Acquisition function	Expected Improvement	Expected Improvement	Lower Confidence Bound
Objective function	Scaled Euclidean distance	Scaled Euclidean distance	Scaled Euclidean distance
Recipes per batch (after initial seed)	1	1	1
Priors	Non-informative	Non-informative	Non-informative

Objective functions here

We now added it to the main text on page 6:

> Algo2 from an open-source software using the Tree-structured Parzen Estimator with an EI acquisition function.[28–30] (3) Algo3 using a Gaussian Process model[31] and a lower confidence bound (LCB) acquisition function. The algorithms all use scaled Euclidean distance as the objective function, and started without any training and using non-informative priors.[32]

Referee 1:

If the distance-to-metric function described in the paper is just used to illustrate progress, but is not what is used by the algorithms, the above critical concern would be substantially reduced with clarification of algorithm setups.

Author Response:

Yes, exactly! We hope our above clarifications are satisfying. We apologize for not making this clearer in our earlier manuscript and are glad to do so now.

Referee 1:

A second (important but not blocking) issue has been half addressed by the revision, but the other half of the question remains. The issue is that both human-specified experiments and human-specified narrower constraints are provided at each transfer point. The added experiment (at transfer point C) shows that if constraints are provided in addition to the sample points, about 5% additional cost savings are achieved. But what if just constraints are provided to the algorithms?

Author Response:

In response, we added a new result to Figure S4 for just constraint with 32 LHC sampling points: see "Constraint, LHC32" in third column below. We also added a fourth column for no human information labelled "No constraint, LHC32," to provide a side-by-side comparison of all four combinations. Here is the new Fig S4 on page 25:

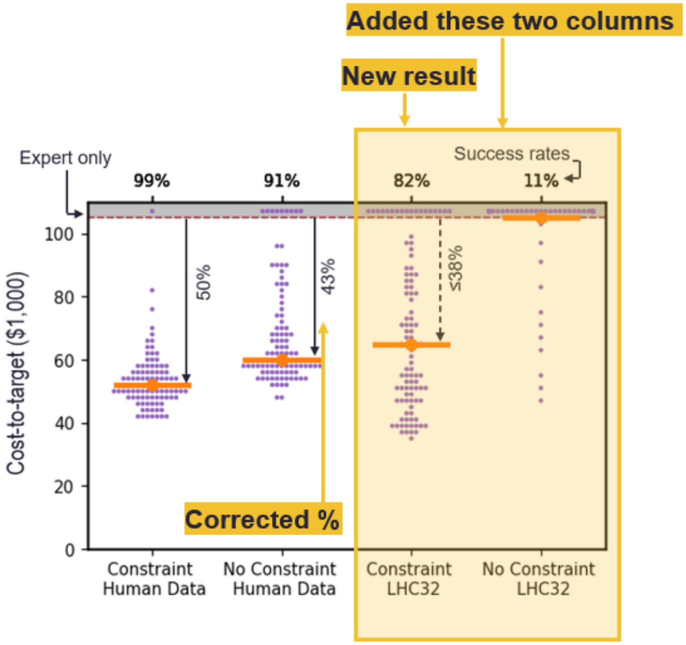

Referee 1:

Maybe that is enough information that, with the dramatically reduced search space, a latin-hypercube sampling (LHS) starting from that information only might better use the available experimental points? (Note that shrinking each parameter space by ½, across 11 parameters, is over a 2000-fold search space reduction, so this could be quite important).

Author Response:

To answer your question, we interpret the new result "Constraint, LHC32" (third column) as there is *more value* in providing the algorithm with 32-points of human data (median cost-to-target of $52,000) than 32-points of LHC random sampling (median cost-to-target >$65,000) when given the constraint.

Note: We write ">$65,000" because we consider "Constraint, LHC32" to be an artificial case. Here, the algorithm is provided the constraint, but we do *not* charge it for the cost of human data to find the constrained regime. This means the cost-to-target for "Constraint, LHC32" could conceivably be even *higher* than $65,000 if we charged for the constraint. This does not affect our current interpretation since a median cost-to-target of $65,000 is already greater than both $60,000 ("No constraint, human

data") and $52,000 ("Constraint, human data"). We indicate this point in the figure with a dotted arrow and explaining in the caption on page 25:

from the human (instead, using 32-point Latin Hypercube random sampling seeds). In the fourth panel, the computer received no information from the human (i.e., transfer point 'no human' in Figure 3c.) The black arrows indicate % cost savings relative to the expert alone, dotted in the third column because we did not charge any cost for access to the constraint. Each dot represents one of 100 independent trajectories. Performance of Algo3 with *both* the expert constrained

---

Referee 1:

Imagine a transfer point "L" (for human-recommended limits) to the left of transfer point "A", but to the right of the "no human" point. One might be L+12 for improved limits and then 12 LHS points; another might be L+32 for algorithm given improved limits and then conducting 32 LHS samples within the smaller space. Note that the motivation for both L+12 and L+32 is that this "starting simulation sample size" is a challenging hyperparameter in surrogate modeling-based methods (like BayesOpt), and considering both of these would also address some concern about neglecting this point in the paper.

Author Response:

As explained above, these cases are artificial as the cost of transferring the constraint is not straightforward. Therefore, we leave the topic of human versus random sampling for further exploration in the future.

Referee 1:

Note also that these are different from transfer point A, where human-provided sample points are also provided, but with the human-induced cost also incurred and the algorithm limited to the human-provided samples; it is possible that random sampling within the reduced space (and especially with more informative distance optimization objective as discussed above) would be better competition to the human sample points. More broadly, such evaluation would help answer the question about relative value of constraints, human-provided, and startup random sampling toward human-algorithm symbiotic improvements, and better disambiguate "human-provided information."

Author Response:

Our new result provides an answer: at transfer point C, we find that human data is more valuable than constraints, and providing constraints with human sampling is more valuable than with LHC initial sampling seed. We thank you for motivating us to do this analysis and including it in the manuscript.

Referee 1:

Side note: the paper is not clear on when or if LHS sampling is done only for the "no human" case, or also in conjunction with transfer points A through E. My interpretation or assumption above, is that in transfers A through E, random sampling startup has been replaced by the human-provided samples. It would be good for the paper to make that explicit or clarify.

Author Response:

Correct, random sampling seed is *not* used with transfer points A to E. We clarified on page 7:

> navigation. Therefore, we decided to test a hybrid strategy where the expert guides the algorithms in a Human First - Computer Last (HF-CL) scenario. In this implementation, instead of random sampling, the expert provides experimental data collected up to a transfer point labeled A through E in Figure 2, along with a constrained search range (Table S3). (For

---

Referee 1:

In two places, the writing seems to suggest that the algorithms choose to ignore information or make decisions, whereas the algorithms had no such choice or decision available to them. One relates to the use of the cross-sectional profiles; was there any kind of setup implemented where the algorithms could have used these profiles, but somehow elected not to and ignored them instead (line 148)?

Author Response:

The algorithms did not have a way to make use of the profile images, data scientists did not program to use these profiles (we hope this will be the focus of a future study). We clarified on page 6:

> In their process games, the algorithms requested one recipe per batch, the default for Bayesian optimizations,[33] and used the output metrics. However, they were not programmed to use the output profile images, and so these were effectively ignored. Trajectories were repeated 100 times for statistical relevancy to account for inherent randomness in cost-to-target due to the probabilistic nature of Bayesian optimization. To save computational time, trajectories were

---

Referee 1:

Similarly, at line 285 the paper says "while the computers requested only one experiment per batch." But the algorithms didn't get to choose: they were inherently set up or limited to only one experiment per batch, so it's odd to use "request" wording to contrast with humans who were enabled to make the batch choice.

Author Response:

We modified the wording on page 6:

In their process games, the algorithms were programmed to utilize one recipe per batch, the default for Bayesian optimizations,[33] and used the output metrics. However, they were not programmed to use the output profile images, and so these were effectively ignored. Trajectories were repeated 100 times for statistical relevancy to account for inherent randomness in cost-to-

---

Referee 1:

One more “cummulative” correction needed – Fig. S2 was corrected but Fig. S3 still has “cummulative” on the horizontal axis.

Author Response:

Thank you for catching this typo as well. Here is the corrected x axis title on Figure S2:

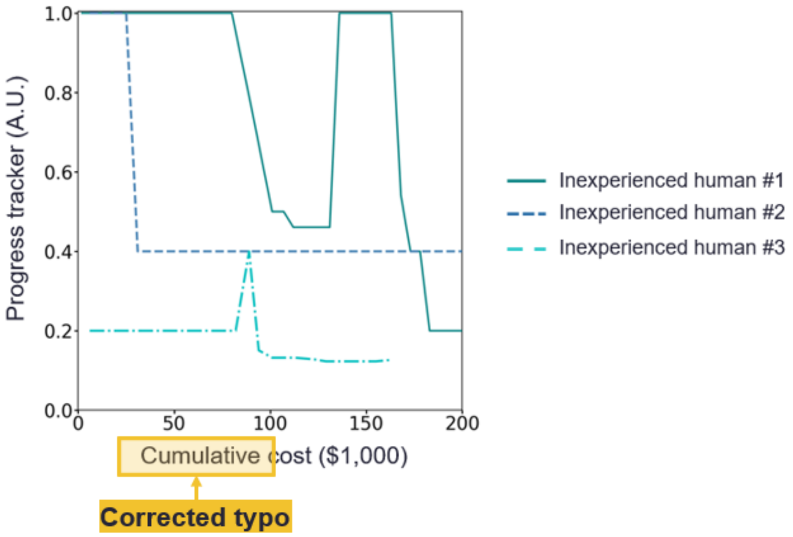

Referee 1:

Labels for human participants in Table S1 appear confused (for JE1 and JE3). I hope that the company is looking to fast-track and promote junior engineer #1; they had impressive engineering performance in this game, both alone and when teamed with a computer algorithm.

Author Response:

Thank you. We are glad the reviewer found these typos.

Referee 1:

Excellent additions by the authors, to provide valuable information about each human and trajectories or behaviors in those cases.

Author Response:

Thank you!

Referee 1:

I am concerned about the validity of the results, related to the particular "distance-to-metric" metric if it or substantially similar framings were used by the algorithms. The authors need to clarify and address this critical concern.

Author Response:

To the first reviewer: We hope we have adequately addressed your remaining concerns. We very much appreciate your input – in this and the previous review, and are extremely grateful for your detailed questions and comments. Thank you again for your time and attention and helping us improve our manuscript.

Referee 2 (note: this is the *other* referee):

Thanks to the authors for the response. Most of the comments have been addressed. The revised manuscript is clearer for readers to understand how humans and AI can work together to accomplish a shared goal.

Author Response:

To the second reviewer: We are glad that we addressed your comments to your satisfaction. Thank you again for your review, your comments, and your support of our study. We are grateful for your time and helping us improve the quality of our manuscript.

Reviewer Reports on the Second Revision:

Referee #1 (Remarks to the Author):

This revision has addressed both major and minor concerns from my previous review. The paper will be a highly valuable contribution to the community.

The new "progress tracker" terminology is good, clarifying and distinguishing from the Euclidean distance objective function. With that (non-truncated) objective, the results presented are persuasive and well-supported by the experiments and analyses.

The two new transfer test cases in Fig. S4 are an excellent addition. The author's interpretation is reasonable: the provision of either data points (without constraints) or data points and constraints, does better on median than just providing constraints to BO. But it's impressive how important those constraints alone are, getting performance that is pretty close to HF-CL. It's also interesting that in this case, there are some fraction of costs-to-target that are even lower than in the HF-CL case. The excellent addition of these test cases and results in S4 will motivate some interesting areas for future research.

Two very minor phrasing issues could be addressed in final manuscript preparation:

Line 265-266: "small number of humans" is a little odd; maybe "small number of test cases" or "small number of combinations"?

Line 287: "while the computers requested only one experiment per batch" would be better as "while the computers were limited to only one experiment per batch" (to be consistent with the other clarifying revisions already made).

Signed by Reviewer: Duane S. Boning

Tracking# 2022-01-00365C

Author Rebuttals to Second Revision:

Request from reviewer

This revision has addressed both major and minor concerns from my previous review. The paper will be a highly valuable contribution to the community.

The new "progress tracker" terminology is good, clarifying and distinguishing from the Euclidean distance objective function. With that (non-truncated) objective, the results presented are persuasive and well-supported by the experiments and analyses.

The two new transfer test cases in Fig. S4 are an excellent addition. The author's interpretation is reasonable: the provision of either data points (without constraints) or data points and constraints, does better on median than just providing constraints to BO. But it's impressive how important those constraints alone are, getting performance that is pretty close to HF-CL. It's also interesting that in this case, there are some fraction of costs-to-target that are even lower than in the HF-CL case. The excellent addition of these test cases and results in S4 will motivate some interesting areas for future research. Two very minor phrasing issues could be addressed in final manuscript preparation:

Line 265-266: "small number of humans" is a little odd; maybe "small number of test cases" or "small number of combinations"?

Line 287: "while the computers requested only one experiment per batch" would be better as "while the computers were limited to only one experiment per batch" (to be consistent with the other clarifying revisions already made).

Author Response:

We are glad that our revision addressed your concerns. We appreciate the time, effort, and kindness that you put into helping improve this manuscript. Thank you!

As for the minor phrasing issues, we agree and modified the text accordingly.
